# Measuring mind wandering with experience sampling during task performance: An item response theory investigation

**DOI:** 10.3758/s13428-024-02446-9

**Published:** 2024-07-25

**Authors:** Anthony P. Zanesco, Nicholas T. Van Dam, Ekaterina Denkova, Amishi P. Jha

**Affiliations:** 1 Department of Psychology, University of Miami, 5665 Ponce de Leon, Coral Gables, FL 33146 USA; 2 School of Psychological Sciences, University of Melbourne, Melbourne, Australia

**Keywords:** Experience sampling, Item response theory, Mind wandering

## Abstract

The tendency for individuals to mind wander is often measured using experience sampling methods in which probe questions embedded within computerized cognitive tasks attempt to catch episodes of off-task thought at random intervals during task performance. However, mind-wandering probe questions and response options are often chosen ad hoc and vary between studies with extant little guidance as to the psychometric consequences of these methodological decisions. In the present study, we examined the psychometric properties of several common approaches for assessing mind wandering using methods from item response theory (IRT). IRT latent modeling demonstrated that measurement information was generally distributed across the range of trait estimates according to when probes were presented in time. Probes presented earlier in time provided more information about individuals with greater tendency to mind wandering than probes presented later. Furthermore, mind-wandering ratings made on a continuous scale or using multiple categorical rating options provided more information about individuals’ latent mind-wandering tendency – across a broader range of the trait continuum – than ratings dichotomized into on-task and off-task categories. In addition, IRT provided evidence that reports of “task-related thoughts” contribute to the task-focused dimension of the construct continuum, providing justification for studies conceptualizing these responses as a kind of task-related focus. Together, we hope these findings will help guide researchers hoping to maximize the measurement precision of their mind wandering assessment procedures.

**Supplementary Information:**

The online version contains supplementary material available at 10.3758/s13428-024-02446-9.

## Introduction

The tendency for individuals’ attention to lapse and wander off-task is commonly assessed using experience sampling methods that aim to capture attentional lapses and task-unrelated thoughts as they occur in the moment (Smallwood & Schooler, 2006, 2015). As such, mind wandering probes are frequently embedded within computerized cognitive tasks to catch mind wandering at random intervals during task performance or throughout daily life using digital applications or devices. It is common for probes to catch individuals’ mind wandering between 30 to 50% of the time in these situations (Kawashima et al., 2023; Killingsworth & Gilbert, 2010; Wong et al., 2022; Zanesco et al., 2024). Although experience sampling methods are ubiquitous in studies of mind wandering, there is nevertheless considerable methodological heterogeneity in the format and wording of mind wandering probe questions (Weinstein, 2018), and few studies have investigated the psychometric consequences of probing methods for the valid and reliable measurement of mind wandering (cf. Kane et al., 2021b; Schubert et al., 2020; Welhaf et al., 2023).

Most commonly, studies of mind wandering employ probes that ask individuals to self-report whether their attention was either focused on-task or focused off-task toward task-unrelated thought. Assessing mind wandering in this manner most frequently involves the use of dichotomous response formats, in which individuals respond by selecting one of two possible options: on-task or off-task (Weinstein, 2018). Another common approach involves presenting multiple categorical response options: individuals select among several options to best describe their current mental state (e.g., either focused on-task, thinking about their task performance, or several categories representing different kinds of task-unrelated thought). These categorical response options are often subsequently dichotomized into on-task and off-task ratings by collapsing over rating categories (e.g., Kane et al., 2016, 2021b). Finally, a growing number of studies have employed probes with multiple polytomous response options arranged along a continuum from on-task to off-task (e.g., ratings from 1 “on-task” to 6 “off-task”; Zanesco et al., 2020).

Individuals’ trait tendency to mind wander is thought to reflect a quantitative continuum of intraindividual differences, with some individuals more prone to mind wander than others (Kane et al., 2017, 2021a; Maillet et al., 2018; McVay et al., 2009; Welhaf et al., 2020). Summary scores based on probe rating responses (e.g., calculated as the mean of probe ratings or the proportion of “off-task” probes for individuals) are therefore commonly used as estimates of an individual’s mind wandering propensity. Importantly, it is an open question whether methodological differences in mind wandering probes (e.g., dichotomous, categorical, or continuous response options) have any consequential influence on the measurement precision of these trait estimates. Indeed, researchers most commonly utilize dichotomous response options, or dichotomize categorical or continuous ratings into on- and off-task categories (Weinstein, 2018), despite well-known concerns regarding the loss of measurement information resulting from dichotomizing continuous data or using dichotomous response options to assess continuous quantitative constructs. This practice can compromise measurement reliability, statistical power, the accuracy and magnitude of individual differences correlations, and the precision of measurements of the underlying latent construct (Cohen, 1983; Fedorov et al., 2009; MacCallum et al., 2002).

On the other hand, researchers may be concerned that individuals find it challenging to distinguish between too many continuous rating options. While using a larger number of options is known to increase the precision, reliability, and, often, validity of instruments (Nunnally, 1967; Weng, 2004), the disadvantage is that individuals might struggle to differentiate increasingly ambiguous option labels or take longer to respond when choosing between many options. Individuals do not always interpret instructions or rating options as researchers might have intended. In addition, the psychometric disadvantages of using dichotomous rating options or continuous scales with fewer rating options can be accommodated by increasing the total number of items to improve the overall precision and reliability of the instrument. As such, methodological choices, regarding the number of items or the number of rating options, may be consequential study design decisions requiring adjudication through empirical investigation (Linacre, 2002). Examination of the consequences of these methodological choices on the probe-caught measurement of mind wandering are therefore warranted.

These considerations may be particularly important for studies of mind wandering in which researchers rely on the fidelity of individuals’ introspection about their fleeting mental states (Seli et al., 2015; Meier, 2018). It has been argued, for example, that individuals are less confident about their off-task ratings when using continuous rating scales (Kane et al., 2021b), and their ratings may be subject to reporting biases resulting from behavioral errors in accompanying cognitive tasks (Head & Helton, 2018). Reducing the total time spent answering probe questions may also be an important consideration because probes in cognitive tasks interrupt the ongoing pace of task performance and increase overall task duration. Probe presentation rate is also negatively associated with overall levels of mind wandering, such that studies with greater probe presentation rate tend to catch fewer episodes of mind wandering (Schubert et al., 2020; Zanesco et al., 2024). More frequent probing might work to entrain individuals’ attention on the task and remind them of their ongoing task-related goals when probes appear.

One approach to evaluate the effect of methodological choices, such as the number of probes or different response options, on probe-caught measurement of mind wandering is through the item response theory (IRT) framework. IRT conceptualizes responses to test or instrument items as observed indicators of an individual’s underlying latent ability (θ) measured across a quantitative continuum. θ is commonly interpreted in a standardized format (e.g., *z*-scores in standard deviation units from the mean), with higher θ values indicative of greater values of the latent trait. Importantly, the many items that make up a test or instrument, or the different response options an individual can provide as answers, contribute differentially to IRT estimates of θ. This is because some items or response options may be more or less likely to be endorsed by individuals of different levels of ability. This can sometimes mean that a test or instrument provides only an adequate amount of measurement precision for scores within a narrow range of the latent continuum because individuals of varying ability are poorly differentiated by the indicators.

Instruments or tests can therefore be designed to provide an optimal amount of information for understanding specific ranges of the trait continuum. Test questions might vary in difficulty or sensitivity so that some questions are more likely to be answered affirmatively by individuals in a specific range. For example, items included in the Mind Excessively Wandering Scale (Mowlem et al., 2019a, b) were designed with the goal of assessing mind wandering in adults with ADHD who are highly prone to mind wander. It is unlikely, however, that most studies of probe-caught mind wandering use probes designed to assess individuals with specific propensities for mind wandering. Instead, it is likely assumed that probes accurately differentiate individuals across all levels of the trait continuum. Probes might better differentiate individuals across varying levels of trait mind wandering by adapting the phrasing of probe questions or response options to optimally distinguish gradations of ability. For example, continuous ratings may provide opportunities for probes to distinguish a wider gradation of ability (e.g., distinguishing individuals prone to report being “mostly off-task” vs. those prone to being “fully off-task”). Information can also be distributed across the trait continuum if responses to probes vary as a function of other task features, such as when they are presented in time – probes later in time being more likely to catch mind wandering than those presented earlier (Brosowsky et al., 2023; Krimsky et al., 2017; Thomson et al., 2014; Zanesco et al., 2020, 2024).

While the format of probe response options may have consequences for the valid and reliable measurement of mind wandering, these methodological choices also have theoretical implications. Indeed, there is considerable debate about which cognitive states or phenomena ought to be considered “mind wandering” (Christoff et al., 2018; Seli et al., 2018b, c). This relates to uncertainty as to which probe questions or response options may best distinguish individuals with differing trait tendencies. For example, ratings of spontaneous versus deliberate mind wandering have been theoretically relevant for understanding the effects of motivation on rates of mind wandering (Seli et al., 2016). Likewise, task-related thought (or “task-related interference”) is often categorized as a task-focused state when ratings are dichotomized into on-task and off-task categories in studies utilizing categorical probe ratings. IRT allows for the evaluation of this assumption by examining whether these “task-related thought” responses are more likely to occur in individuals with less propensity for mind wandering.

There are also open theoretical questions as to whether the state of mind wandering at any one moment is best understood as a dichotomous experience or exists along a quantitative continuum. For example, researchers may object to the use of continuous response options because they believe task-related focus and off-task focus exist as psychologically distinct taxa rather than gradations along a construct continuum (see for further discussion, Kane et al., 2021b; Robison et al., 2019; Schad et al., 2012; Seli et al., 2018a; Zanesco et al., 2020), or that individuals could not accurately distinguish between experiential gradations were they to exist. Although individuals utilize a range of continuous responses when provided the option (e.g., Seli et al., 2018a; Zanesco et al., 2020), variability in continuous ratings can also result from measurement error arising from a dichotomous latent construct. Psychometric modeling has found that “all-or-nothing” attentional lapses contribute to performance deficits in sustained attention tasks (Gyles et al., 2023; McCarley & Yamani, 2021; Román-Caballero et al., 2023), providing some justification for conceptualizing and measuring episodes of mind wandering as a dichotomous mental state (i.e., on-task or off-task).

In the present study, we utilize IRT to evaluate the measurement properties of different mind wandering probe rating methods utilizing dichotomous, categorical, or continuous response options. We compared the level of measurement precision provided by probes across the general latent trait continuum when ratings are made on continuous, polytomous rating scales (i.e., ratings from 1 “on-task” to 6 “off-task”) to those made using dichotomous ratings (i.e., ratings of “on-task” or “off-task”). Importantly, IRT methods require large sample sizes: we rely on three previously published data sets that use larger sample sizes. These data represent several common approaches used to measure mind wandering using dichotomous, categorical, and continuous response options among several cognitive tasks. Our hope is that we might provide new considerations and guidance to improve the precision, reliability, and validity of experience sampling approaches for the assessment of mind wandering.

## Methods

Deidentified participant data were acquired from online data-sharing repositories via the Open Science Framework. Participants in all studies provided informed consent in accordance with the Institutional Review Board of the institution where the study was conducted. Secondary analyses of these data used the same participant exclusions and outlier treatment as reported previously (i.e., Goller et al., 2020; Kane et al., 2016; Zanesco et al., 2020). No other openly available data were examined as part of the present investigation. Relevant details and procedures from these studies are described below in brief and summarized in Table 1.

**Table 1:** Summary of study and mind wandering probe characteristics

Study	Task	*N*	Num. Probes	Probe type	Mean (*SD*)
Zanesco et al. (2020)	SART	514	28	Continuous (1–6)	1.864 (1.034)
Goller et al. (2020)	SART	355	36	Continuous (1–5)	2.508 (0.880)
Kane et al. (2016)	SART	526	45	Categorical (1–8)	0.510 (0.243)
	Arrow Flanker	479	20	Categorical (1–8)	0.490 (0.307)
	Number Stroop	478	20	Categorical (1–8)	0.451 (0.310)
	Letter Flanker	462	12	Categorical (1–8)	0.586 (0.263)
	*N*-Back	461	15	Categorical (1–8)	0.424 (0.313)

The characteristics of included studies are summarized above. Five different tasks were employed across studies, each with embedded experience sampling mind wandering probes. Tasks include the Sustained Attention to Response Task (SART), an Arrow Flanker, Number Stroop, Letter Flanker, and *N*-Back task. The sample size and number of embedded probes are given. Probes were delivered in polytomous (continuous) rating formats or in categorical format. The number of response options are given in parentheses. The mean rating (*SD*) from continuous scales is provided for Zanesco et al. (2020) and Goller et al. (2020), as well as the mean proportion of probes categorized as “off-task” for tasks from Kane et al. (2016)

### Zanesco et al. (2020)

Zanesco et al. (2020) assessed military service members (*N* = 537, *M* age = 28.25 years, *SD* = 8.071, range, 18–54) who were recruited as part of a series of studies investigating the implementation of cognitive resilience interventions in the military. These participants completed the Sustained Attention to Response Task (SART; Robertson et al., 1997) as part of a battery of cognitive behavioral tasks and questionnaires administered prior to random assignment to any intervention arm. During the SART, participants were instructed to press the spacebar for frequent non-target stimuli and refrain from pressing the spacebar in response to infrequent target stimuli. Twenty-eight pairs of probe questions were quasi-randomly distributed throughout the task but were presented on the same relative trials for all participants. Participants were instructed that probes will occasionally ask about the focus of their attention. For each of the 28 pairs of probes, participants responded to two questions: the first question (attentional focus probe) asked, “Where was your attention focused just before the probe?” with participants responding using a six-point scale ranging from 1 (on-task) to 6 (off-task). The second question (awareness probe) asked, “How aware were you of where your attention was?” with participants responding from 1 (aware) to 6 (unaware). Only the attentional focus probe (first probe question) was examined herein.

### Goller et al. (2020)

Goller et al. (2020) assessed undergraduate student participants (*N* = 371, *M* age = 19 years, *SD* = 1.5) recruited from Western Carolina University. These participants completed the SART as part of a battery of cognitive behavioral tasks and questionnaires. Thirty-six pairs of mind wandering probe questions were randomly presented throughout the task. The first question asked about the current content of individuals’ thought (i.e., “What were you just thinking about?”). There were six categorical response options that included: (1) task-related thoughts pertaining to the current task; (2) positive task-related evaluative thoughts; (3) negative task-related evaluative thoughts; (4) neutral task-unrelated thoughts; (5) positive task-unrelated thoughts; and (6) negative task-unrelated thoughts. Importantly, a second probe question immediately followed the first, which asked how on-task or off-task individuals were according to a five-point scale from 1 (completely on-task) to 5 (completely off-task). Only the attentional focus probe (second probe question) was examined herein.

### Kane et al. (2016)

Kane et al. (2016) assessed undergraduate student participants (*N* = 545, age range, 18–35) who were recruited from the University of North Carolina at Greensboro (UNCG). Participants completed several cognitive tasks at three separate assessment sessions. At the first assessment, a semantic version of the SART was administered followed later by a Letter Flanker task. At the second assessment, an Arrow Flanker task was administered followed by a Number Stroop task. A 2-Back working memory (*N*-Back) task was administered at the third assessment. Complete details regarding study procedures are reported elsewhere (Kane et al., 2016; Welhaf et al., 2020).

Categorical, content-based probes were randomly presented throughout each task. Forty-five probes occurred randomly throughout the SART (7% of all trials), whereas 20 occurred in the Arrow Flanker (4.2% of block-1 trials and 16.6% of block-2 trials), 12 in the Letter Flanker (8.3% of trials), 20 in the Number Stroop (13% of block-2 trials), and 15 in the *N*-Back task (6.3% of trials). For each mind wandering probe, participants selected among eight possible options to describe their current focus or thoughts: focused “on-task” (option 1); thoughts directed towards “task performance and evaluation” (option 2); thoughts about normal life concerns and “everyday things” (option 3); thoughts about one’s current physical, cognitive or emotional “state of being” (option 4); thoughts about “personal worries” (option 5); fantastical thoughts and “daydreams” (option 6); thoughts about stimuli in the “external environment” (option 7); and “other” thoughts that were not described by other categories (option 8). The eight probe rating categories were dichotomized into on-task (ratings of 1 “on-task” and 2 “task-related thoughts”) and off-task categories (ratings 3 through 8), in line with how these ratings have been categorized in prior studies (e.g., Kane et al., 2016, 2021b; Welhaf et al., 2020).

### Item response theory models

We applied dichotomous two-parameter logistic (2PL) models, polytomous two-parameter graded response models (2P GRM), and categorical two-parameter nominal response models (2P NRM) to mind wandering probe rating data using the *mirt* package in R (Chalmers, 2012). 2PL models were used for probe rating data with dichotomous response formats (e.g., probes dichotomized into either “on-task” or “off-task”). Graded response models (2P GRM) were used for data with ordinal, polytomous response formats (e.g., a six-point rating scale from 1 “on-task” to 6 “off-task”), whereas the nominal response model (2P NRM) was used for data with multiple categorical response options (e.g., categorical rating options such as “on-task” or “daydreams”). Potential differences in probe timing were ignored for these analyses. Each of these models aim to estimate how the different indicators that make up an instrument (i.e., mind wandering probe ratings) contribute to latent trait (θ) estimates. θ values can be interpreted as standardized values (i.e., *z*-scores) ranging along a latent continuum of ability with θ = 0 equal to mean ability. In the present case, individuals with higher θ values are more prone to mind wander.

Response options and indicators can vary in their difficulty parameters (*b*_i_), which describe the point along the trait continuum where there is 50% probability of selecting a given response option. Higher *b*_i_ values indicate that individuals exhibiting greater levels of latent mind wandering are more likely to select a given response option. The second parameter is the item discrimination parameter (*a*_*i*_), which reflects how well each probe differentiates between individuals at the point on the construct continuum where the curve of the response option is anchored. Higher values of *a*_*i*_ indicate that a particular indicator is better at differentiating individuals on either side of the item difficulty (*b*_i_) threshold. For polytomous models of ordinal response options, difficulty parameters (*b*_i_) are estimated for each response option. Unlike two-parameter models, which allow both item difficulty and discrimination to vary across indicators, one-parameter models constrain item discrimination (*a*_*i*_) to be equal across items. For graded and dichotomized data, we evaluated the relative fit of two-parameter models (2PL and 2P GRM) to one-parameter models (1PL and 1P GRM) using a log-likelihood ratio test.

For nominal response models (i.e., 2P NRM) of multiple categorical response options (Bock, 1997), both slope (*a*_i_) and intercept parameters (*c*_i_) are freely estimated for each of the response options. The interpretation of these parameters also differs from other item response models. The intercept parameter (*c*) represents the probability of responding using a particular response option at θ = 0 (in log odds), whereas the slope parameter (*a*) represents the rate of change in probability across the range of θs (i.e., change in log odds). Negative slope parameters indicate that a particular response option is less likely to be selected at increasing levels of θ, whereas positive slope parameters indicate that the response option is more likely to be selected at increasing levels of θ.

Together, item parameters characterize the probability of individuals endorsing particular response options at different levels along the latent trait continuum. This can be visualized through item characteristic curves, which depict the probabilities of endorsing specific response options across levels of the latent trait continuum for the many indicators of an instrument or test. In addition, these parameters provide *information* about how well each indicator contributes to the measurement of θ across levels of the trait continuum. Greater total information, across a broad range of the continuum, can be a desirable feature for an instrument because this indicates that all levels of the trait continuum are measured with adequate precision. The total information of an instrument or test is directly related to the measurement error surrounding estimates of θ.

## Results

Table 1 summarizes the methodological features of studies included in analyses, including the sample size, the cognitive task completed by participants, and the type and number of probes presented during each task.

### Item response theory analysis of Zanesco et al. (2020)

#### Graded response model for continuous ratings

**Fig. 1:**
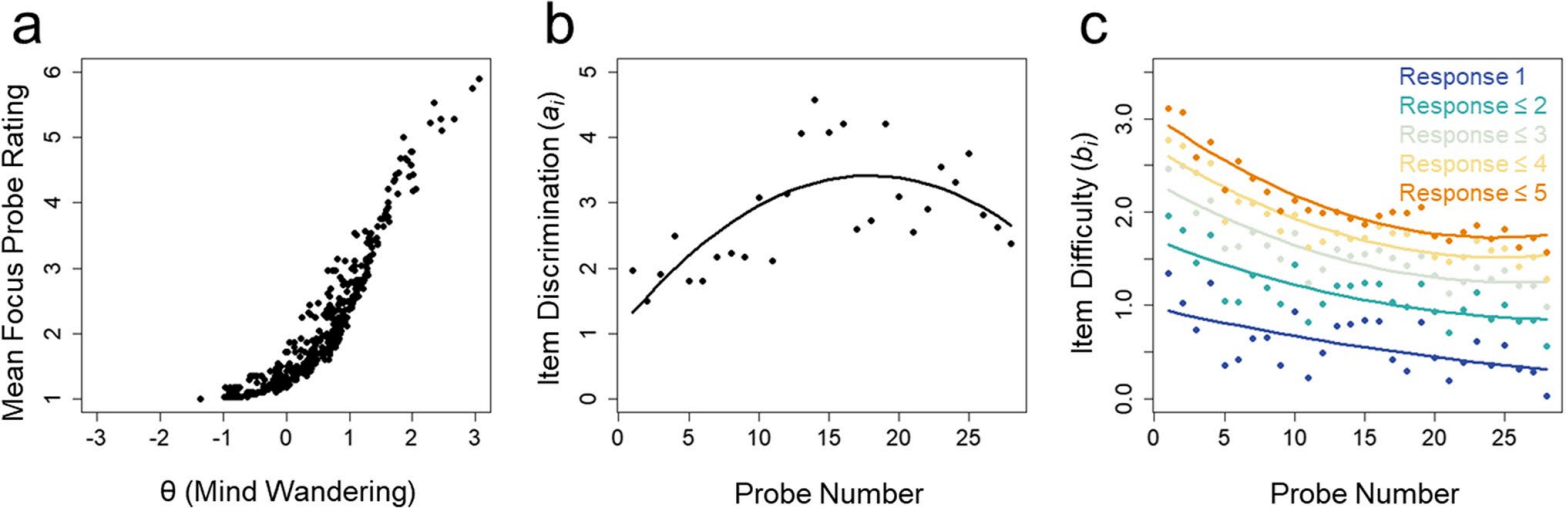
Parameters from graded response models are provided for data from Zanesco et al. (2020). **a** All participants’ (*N* = 514) mean focus probe ratings (from 1 “on-task” to 6 “off-task”) alongside their corresponding θ values estimated based on the graded response model. **b** Discrimination values (*a*_*i*_) of the 28 sequential probes delivered during the SART. The *black line* depicts the polynomial quadratic trajectory of the values. **c** Item difficulty values (*b*_*i*_) of the 28 sequential probes for each response category. *Solid lines* depict the polynomial quadratic trajectories of the values

To evaluate the measurement of latent mind wandering tendency by mind wandering probes, a polytomous one-parameter graded response model (1P GRM) and polytomous two-parameter graded response model (2P GRM) were fit to data from 514 individuals who responded to 28 mind wandering probes embedded within the SART. Individuals rated the degree of their on-task or off-task focus on a six-point ordinal scale. As shown in Table 2, the 2P GRM fit the data significantly better than the 1P GRM. Accordingly, the results of the two-parameter model are reported here. Item discrimination and difficulty parameters for all probes are provided in Supplementary Table 1. Individuals’ mean mind wandering probe ratings are shown alongside their θ values estimated from the 2P GRM in Fig. 1A.

**Table 2:** Model fit of the one- and two-parameter graded response model

Study	Model	AIC	BIC	Log-Lik.	χ^2^	*df*	*p* value
Zanesco et al. (2020)	1P GRM	25496.7	26094.9	– 12607.4			
	2P GRM	25237.9	25950.6	– 12451.0	312.8	27	< .001
Goller et al. (2020)	1P GRM	31751.2	32312.7	– 15730.6			
	2P GRM	31536.8	32233.8	– 15588.4	284.4	35	< .001

Model comparisons of one-parameter graded response models (1P GRMs) and two-parameter graded response models (2P GRMs) from Zanesco et al. (2020) and Goller et al. (2020) are provided alongside the Akaike Information Criterion (AIC), Bayesian Information Criterion (BIC), and log-likelihood of each model. The χ2 log-likelihood ratio test between models is given with degrees of freedom (*df*) and accompanying *p* values

**Fig. 2:**
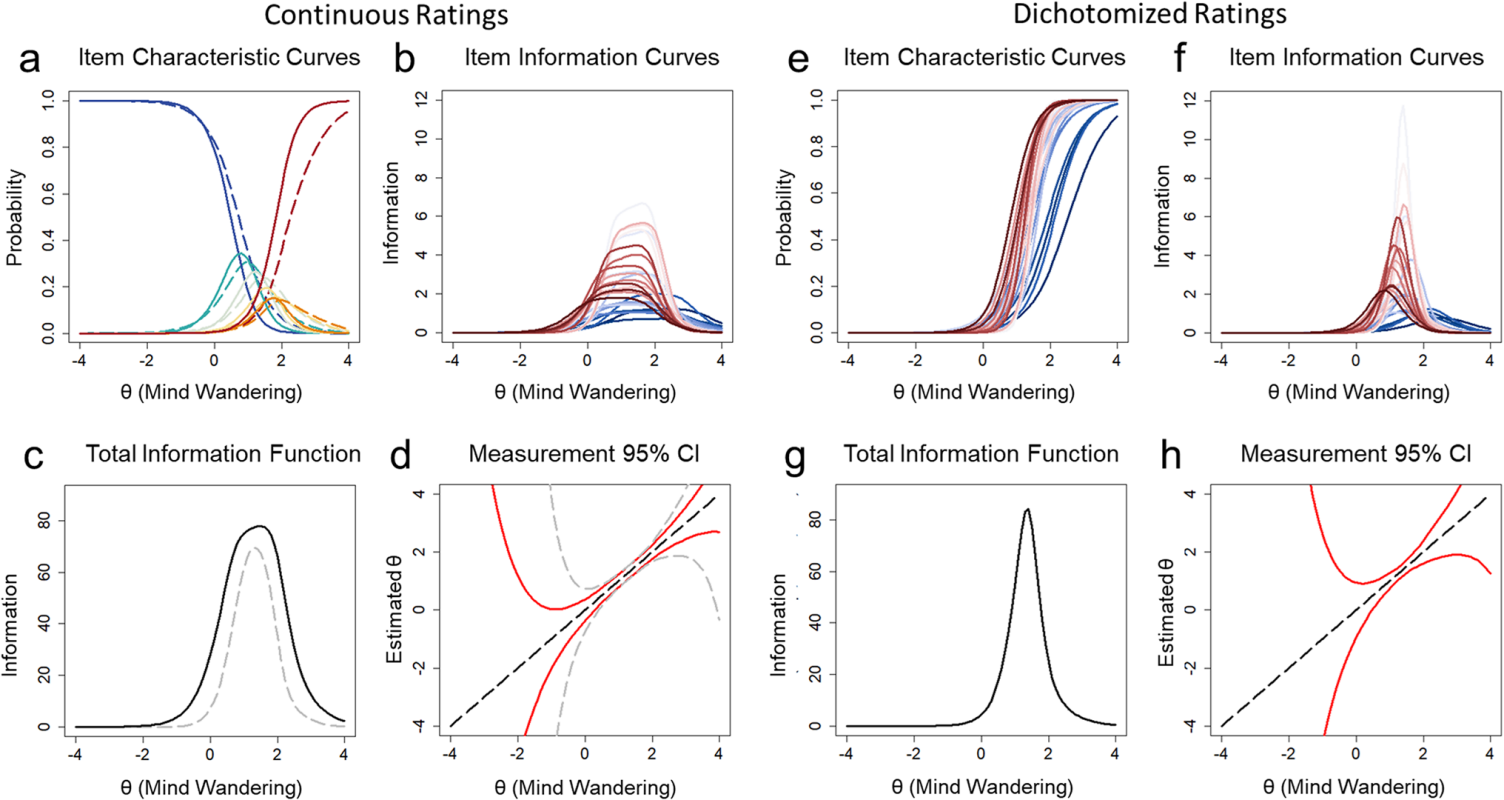
Panels **a**–**d** reflect item response characteristics of continuous response option data while panels **e**–**h** reflect continuous data converted to dichotomized responses. **a** Average item characteristic curves based on the two-parameter graded response model (2P GRM) for all six probe response options from Zanesco et al. (2020). Each *curve* represents the probability of a participant endorsing a particular response option at all levels of trait mind wandering (θ) on the *x*-axis. Higher θ values are indicative of greater trait mind wandering tendency. Separate curves represent response options 1 through 6. The *dashed curves* represent the average of item characteristic curves for probes 1 through 14 (early probes), whereas *solid curves* represent the average for probes 15 through 28 (later probes). Item discrimination (*a*_*i*_) and difficulty (*b*_*i*_) parameters from the models determine the location and slope of each curve. **b** Item information curves for all probes. Item characteristic curves and information curves range in colors from *dark blue* for early probes to *dark red* for later probes. Panel **c** displays the total information, reflecting the sum of information provided by all probes and response options. The *dashed grey line* depicts the total information provided when ratings of 1 “on-task” and ratings of 6 “off-task” are excluded. **c** Measurement precision of estimates of trait mind wandering (θ) shown based on the total information. The *red line* depicts the 95% CI around θ estimates at all levels of θ. The *dashed grey line* **(d)** depicts the 95% CI around θ estimates based on item information provided when ratings of 1 “on-task” and ratings of 6 “off-task” are excluded. **e** Item characteristic curves based on the 2PL model shown for the probes when dichotomized. *Curves* represent the probability of a participant endorsing an “off-task” response at all levels of trait mind wandering (θ). Panels** f**–**h** are analogs of panels **b**–**d** for the dichotomized data

Item difficulty parameters (*b*_*i*_; the θ value where the probability of selecting a particular response option is 50%) were primarily distributed across the upper range of θ values. Item discrimination parameters (*a*_*i*_; the ability of the items to differentiate between individuals with varying levels of θ) ranged from 1.50 to 4.58. The temporal distribution of discrimination and difficulty parameters suggested that probes presented earlier in the task had lower discrimination and higher difficulty values than later probes (see Fig. 1B and C). Difficulty and discrimination values reached an asymptote toward the middle of the task. This distribution was confirmed by a polynomial quadratic trend fit to the discrimination values across probes, which was significant and explained 50% of the variance, *R*^2^ = 0.498, *F*(2,25) = 12.39, *p* < .001 (see Fig. 1B). Quadratic trends were also significant for difficulty values for probe rating responses ≤ 3, *R*^2^ = 0.760, *F*(2,25) = 39.63, *p* < .001, responses ≤ 4, *R*^2^ = 0.836, *F*(2,25) = 63.54, *p* < .001, and responses ≤ 5, *R*^2^ = 0.881, *F*(2,25) = 92.12, *p* < .001, whereas linear trends described responses of 1, *R*^2^ = 0.354, *F*(1,26) = 14.22, *p* < .001, and responses ≤ 2, *R*^2^ = 0.558, *F*(1,26) = 32.85, *p* < .001 (see Fig. 1C). These trends in item parameters were also evident in the item characteristic curves for the different response options (see Fig. 2A). The mean item characteristic curves of early probes (Fig. 2A, dashed lines) were distributed towards the higher end of θ values across response options compared to the mean curves of later probes, suggesting that early probes primarily provide information about the higher end of the spectrum of latent mind wandering tendency.

Item information is provided for each probe in Fig. 2B, and the total test information from all 28 mind wandering probes (total information = 185.93) is shown in Fig. 2C. Only 36.27% of total information was contained within θ = – 1 to θ = 1. This distribution of test information resulted in a large degree of precision in measuring latent mind wandering tendency at the mean and higher range of the trait continuum (i.e., θs ≥ 0). Figure 2D depicts the 95% CIs around θ estimates at all levels of θ. Measurement was therefore more precise at average θ values and above with 95% CI ranges of 0.739, 0.453, 0.483, and 1.117 for θ = 0, 1, 2, and 3, respectively. In contrast, measurement precision was poor below the mean with 95% CI ranges of 2.074, 6.345, and 18.285 for θ = – 1, – 2, and – 3, respectively.

Importantly, ratings made on intermediate positions of the continuous rating scale (ratings of 2, 3, 4, or 5) provided substantial information about latent mind wandering tendency: 57.42% of the total test information from probes. Figure 2C (dashed grey lines) shows the information provided when ratings of 1 “on-task” and 6 “off-task” are excluded relative to the total information. Measurement was therefore more imprecise when information is provided by only ratings of 1 “on-task” and 6 “off-task” (see Fig. 2D, dashed grey lines). This suggests that intermediate ratings on the continuous scale provide incremental improvements in the measurement of latent mind wandering tendency over and above ratings of 1 “on-task” and 6 “off-task” alone.

#### Two-parameter logistic model for dichotomized ratings

We next dichotomized the continuous ratings into on- and off-task categories by classifying ratings of 1, 2, and 3 into on-task episodes and ratings of 4, 5, and 6 into off-task episodes. A two-parameter logistic (2PL) model was fit to the dichotomous ratings and item discrimination and difficulty parameters are provided in Supplementary Table 2. Results from an model with an alternative categorizing scheme classifying ratings of 1 into “on-task” and ratings > 1 into “off-task” are presented in the Supplementary Materials. Item difficulty parameters (*b*_*i*_) ranged from 0.84 to 2.55, whereas item discrimination parameters (*a*_*i*_) ranged from 1.77 to 6.87. Early probes had lower discrimination and higher difficulty values than later probes. Figure 2E depicts the item characteristic curves for all probes based on the 2PL model parameters. The item characteristic curves of early probes (Fig. 2E, blue lines) were distributed towards the higher end of θ values compared to later probes (red lines).

Item information was high in a narrow range of θ values (see Fig. 2F), with early probes providing more information at the higher end of the latent continuum. The total test information from all 28 mind wandering probes (total information = 96.39) is shown in Fig. 2G. This represents a 48.16% reduction in information relative to the results of the two-parameter graded response model using continuous ratings (see Fig. 2G compared to 2C). 23.55% of the total information from the 2PL model was contained within θ = – 1 to θ = 1. Figure 2H depicts the 95% CIs around θ estimates at all levels of θ. Measurement was less precise below θ = 0 and above θ = 2. Thus, when continuous ratings were dichotomized into on-task and off-task states, there was a large loss in information from the lower and higher range of the trait continuum.

### Item response theory analysis of Goller et al. (2020)

#### Graded response model for continuous ratings

**Fig. 3:**
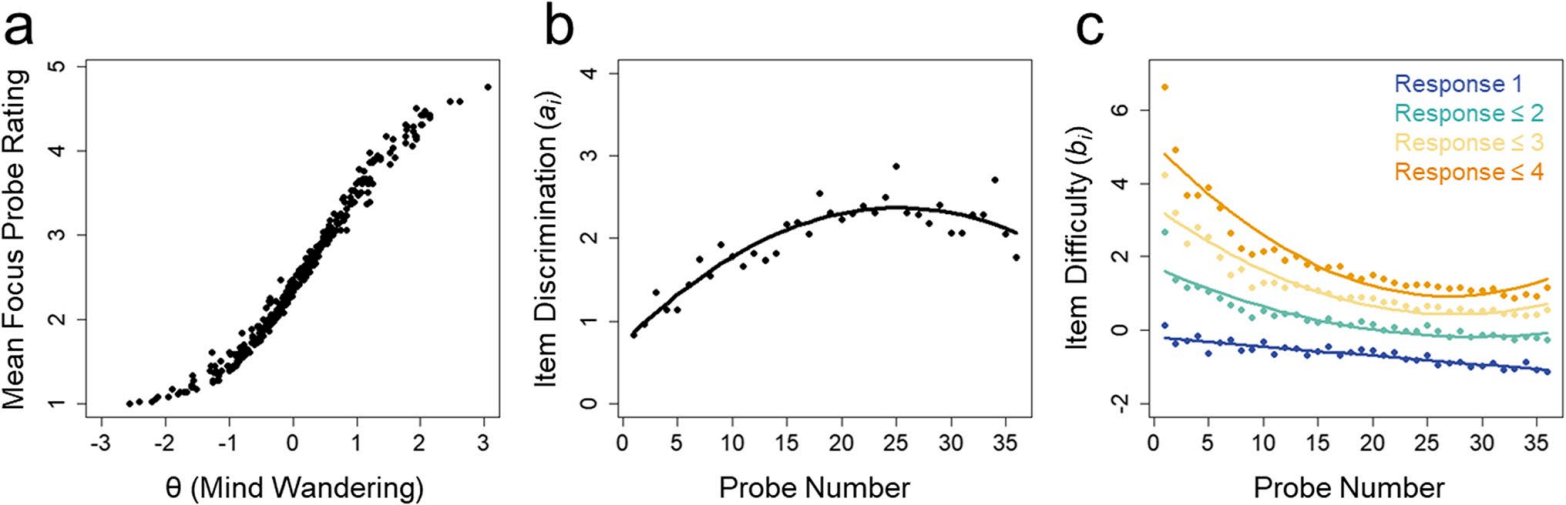
Parameters from graded response models are provided for data from Goller et al. (2020). **a** All participants’ (*N* = 355) mean focus probe ratings (from 1 “on-task” to 5 “off-task”) alongside their corresponding θ values estimated based on the graded response model. **b** Discrimination values (*a*_*i*_) of the 36 sequential probes delivered during the SART. The *black line* depicts the polynomial quadratic trajectory of the values. **c** Item difficulty values (*b*_*i*_) of the 36 sequential probes for each response category. *Solid lines* depict the polynomial quadratic trajectories of the values

A one-parameter graded response model (1P GRM) and two-parameter graded response model (2P GRM) were fit to data from 355 individuals who responded to 36 mind wandering probes embedded within the SART. Individuals rated the degree of their on-task or off-task focus on a five-point ordinal scale. As shown in Table 2, the 2P GRM fit the data significantly better than the 1P GRM. The results of the 2P GRM are therefore described below, and item discrimination and difficulty parameters are provided in Supplementary Table 3. Individuals’ mean mind wandering probe ratings are shown alongside their θ values estimated from the 2P GRM in Fig. 3A.

**Fig. 4:**
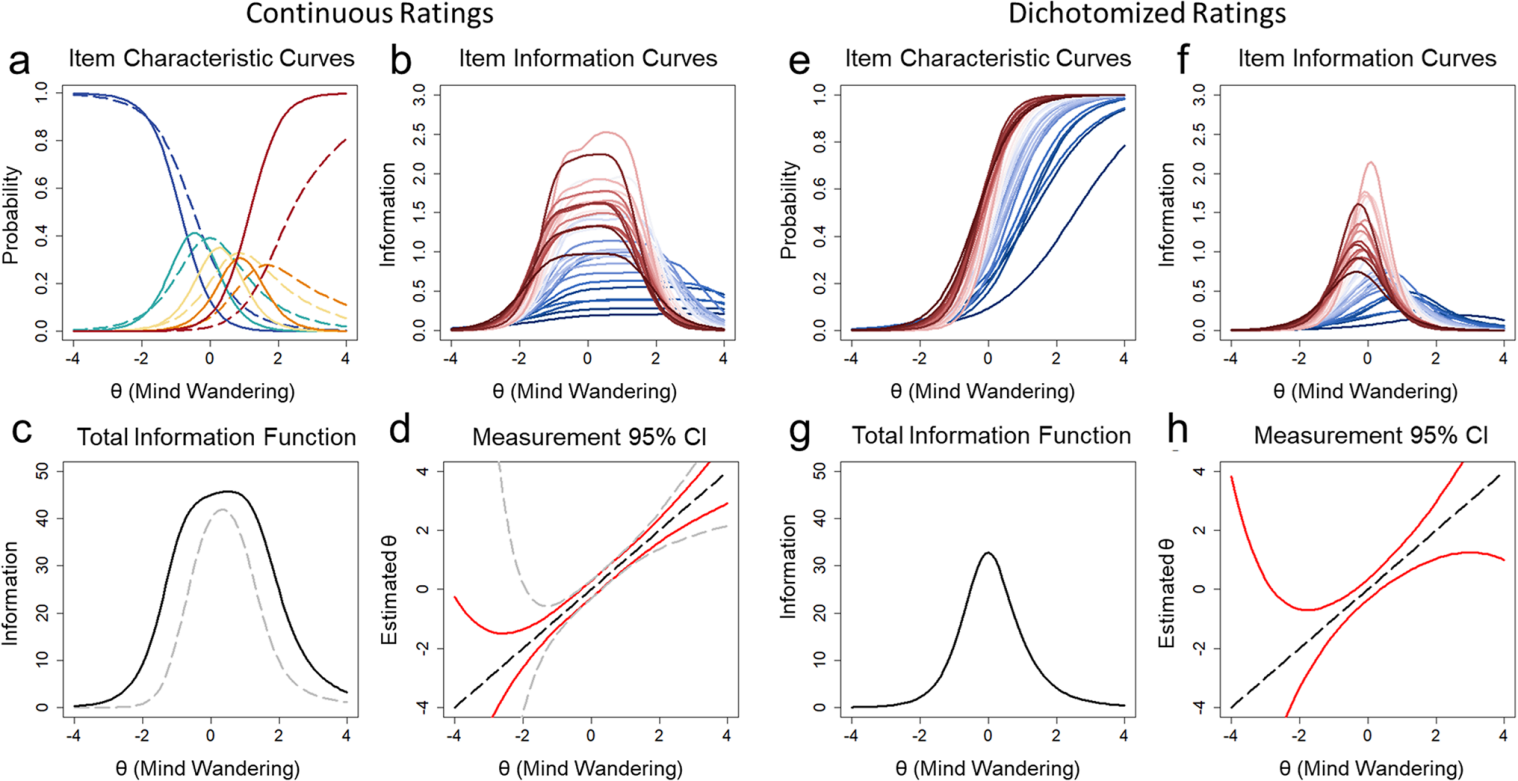
Panels **a**–**d** reflect item response characteristics of continuous response option data while panels **e**–**h** reflect continuous data converted to dichotomized responses. **a** The average item characteristic curves based on the graded response model for all five probe response options from Goller et al. (2020). *Separate curves* represent response options 1 through 5. The *dashed curves* represent the average of item characteristic curves for probes 1 through 18 (early probes), whereas *solid curves* represent the average of probes 19 through 36 (later probes). **b** Item information curves for all probes. **c** The total information. The *dashed grey line* depicts the total information provided when ratings of 1 “on-task” and ratings of 5 “off-task” are excluded. **d** Measurement precision of estimates of trait mind wandering (θ) shown based on the total information. The *red line* depicts the 95% CI around θ estimates at all levels of θ, and the *dashed grey line* depicts the 95% CI based on item information provided when ratings of 1 “on-task” and ratings of 5 “off-task” are excluded. Panels **f**–**h** are analogs of panels **b**–**d** for the dichotomized data

Item difficulty parameters (*b*_*i*_) were distributed across a large range of θ values, while item discrimination parameters (*a*_*i*_) ranged from 0.84 to 2.54 (Supplementary Table 3). Probes presented earlier in the task appeared to have lower discrimination and higher difficulty values than later probes (see Fig. 3B, C). Difficulty and discrimination values reached an asymptote toward the middle of the task. This distribution was confirmed by a polynomial quadratic trend fit to the discrimination values across probes, which was significant and explained 84% of the variance, *R*^2^ = 0.839, *F*(2,33) = 86.08, *p* < .001 (see Fig. 3B). Quadratic trends were also significant for difficulty values for probe rating responses ≤ 2, *R*^2^ = 0.851, *F*(2,33) = 94.46, *p* < .001, responses ≤ 3, *R*^2^ = 0.896, *F*(2,33) = 142.60, *p* < .001, and responses ≤ 4, *R*^2^ = 0.876, *F*(2,33) = 116.50, *p* < .001, whereas linear trends described responses of 1, *R*^2^ = 0.809, *F*(1,34) = 144.40, *p* < .001 (see Fig. 3C). These trends in item parameters were also evident in the item characteristic curves (Fig. 4A).

Item information was high across the range of θ values (see Fig. 4B). But early probes tended to provide more information in the higher end of θ values than later probes. The total test information from all 36 mind wandering probes (total information = 174.42) is shown in Fig. 4C and was broadly distributed across the range of θ values. Only 49.99% of the total information was contained within θ = – 1 to θ = 1. Figure 4D (red lines) depicts the 95% CIs around θ estimates at all levels of θ. Measurement was more precise for individuals with latent mind wandering at the mean and 1 or 2 standard deviations above and below the mean with 95% CI ranges around θ estimates of 0.588 and 0.789 for θ = 1 and 2, and 0.664 and 1.288 for θ = – 1 and – 2, respectively. Information of probes was poor for differentiating individuals 3 standard deviations above and below the mean with 95% CI ranges of 1.352 and 3.204, respectively.

Ratings made on intermediate positions of the continuous rating scale (ratings of 2, 3, or 4) provided substantial information about latent mind wandering tendency: 60.01% of the total test information from probes. Figure 4C (dashed grey lines) shows the information provided when ratings of 1 “on-task” and 5 “off-task” are excluded relative to the total information. Measurement was therefore more imprecise when information is provided by only ratings of 1 “on-task” and 5 “off-task” (see Fig. 4D, dashed grey lines).

#### Two-parameter logistic model for dichotomized ratings

We next dichotomized the continuous ratings into on- and off-task categories by classifying ratings of 1 and 2 into on-task episodes and ratings of 3, 4, and 5 into off-task episodes. A two-parameter logistic (2PL) model was fit to the data and item discrimination and difficulty parameters are provided in total in Supplementary Table 4. Results from an alternative categorizing scheme classifying ratings of 1 into “on-task” and ratings > 1 into “off-task” are presented in the Supplementary Materials. Item difficulty parameters (*b*_*i*_) ranged from – 0.34 to 2.55, and item discrimination parameters (*a*_*i*_) ranged from 0.89 to 2.93. Early probes had lower discrimination and higher difficulty values than later probes. Figure 4E depicts the item characteristic curves for all probes based on the 2PL model parameters. The item characteristic curves of early probes (Fig. 4E, blue lines) were distributed towards the higher end of θ values.

Item information was high in a narrow range of θ values (see Fig. 4F), with earlier probes (blue lines) being more distributed towards the higher end of the trait continuum. The total test information from all 38 mind wandering probes (total information = 69.03) is shown in Fig. 4G and was more narrowly distributed around the mean θ. This represents a 60.42% reduction in information relative to the results of the 2P GRM (see Fig. 4G compared to 4C). 71.93% of total information from the 2PL model was contained within θ = – 1 to θ = 1. Figure 4H depicts the 95% CIs around θ estimates at all levels of θ. Measurement was more imprecise at differentiating individuals 1, 2, or 3 standard deviations above or below the mean. Information was therefore lost from the lower and higher range of the trait continuum when continuous ratings were dichotomized into on-task and off-task states.

### Item response theory analysis of Kane et al. (2016)

#### Two-parameter nominal response model for categorical ratings

A two-parameter nominal response model (2P NRM) was fit to the data from individuals who completed five cognitive tasks with embedded mind wandering probes. The number of individuals who completed each task and number of probes presented in each task are summarized in Table 1. For probes, individuals reported the content of their current focus using eight possible categorical response options. The results of the 2P NRM are described here, and item slope (*a*_*i*_) and intercept (*c*_*i*_) parameters are provided for all tasks in Supplementary Materials Tables 5–9.

**Fig. 5:**
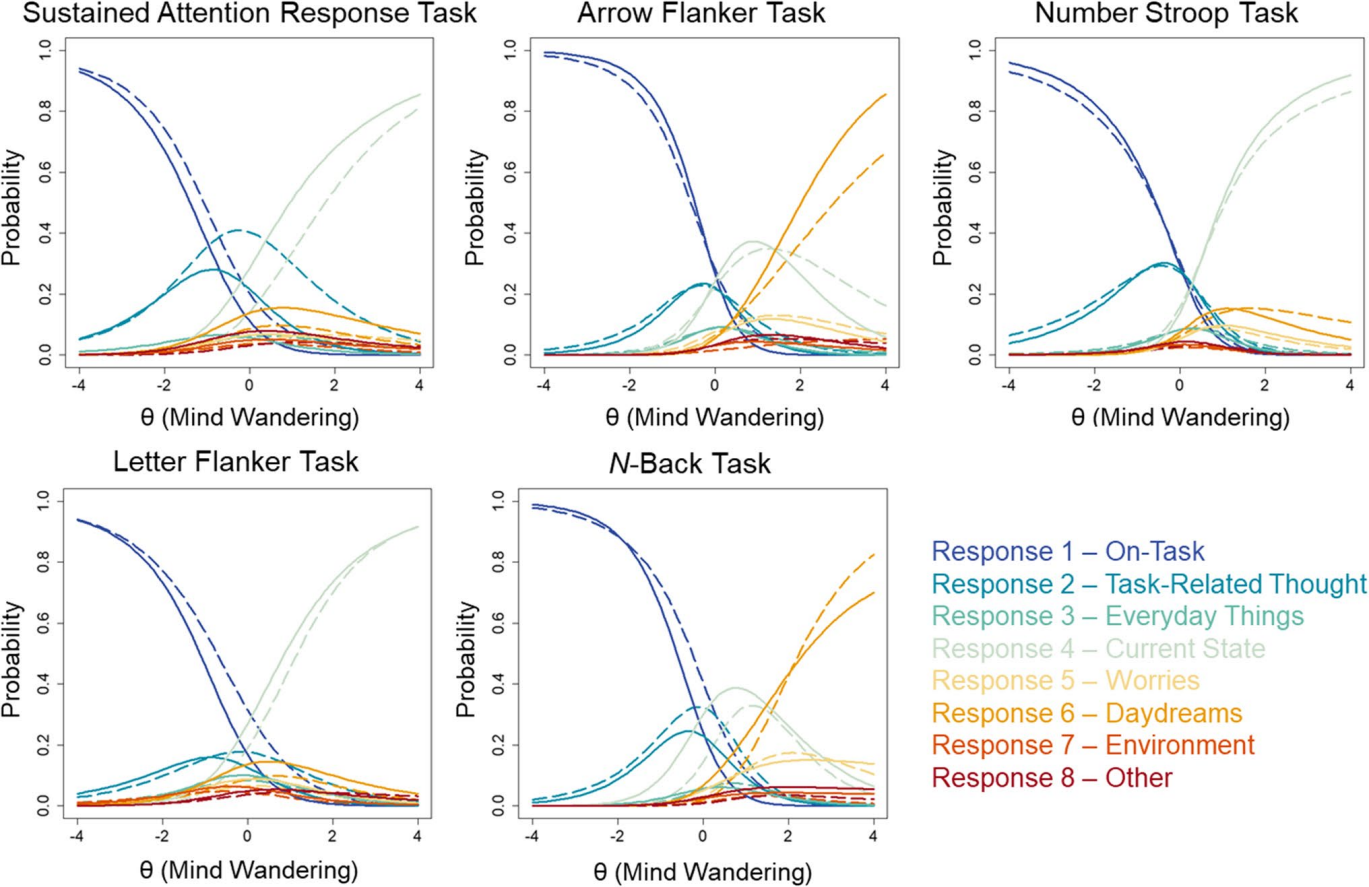
Item characteristic curves are shown based on two-parameter nominal response models (2P NRM) of probes for all five cognitive tasks from Kane et al. (2016). Each *curve* represents the probability of a participant endorsing a particular response option at all levels of trait mind wandering (θ). The *dashed curves* represent the average of item characteristic curves for early probes (SART: 1 through 23; Arrow Flanker: 1 through 10; Stroop: 1 through 10; Letter Flanker: 1 through 6; and *N*-Back: 1 through 8), whereas *solid curves* represent the average of the remaining, later probes

As seen from the item characteristic curves for all probes based on the 2P NRM (see Fig. 5), the probability of responding was high for only specific response options. Response category 1 “on-task”, 2 “task-related thought”, 4 “current state”, and 6 “daydreams” had the largest intercept parameters and therefore were the response options most likely to be utilized by participants. Nominal response item difficulty parameters (*c*_*i*_) were distributed towards the lower end of θ values for response options 1 “on-task” and 2 “task-related thought”, for all tasks. In contrast, all other response options had difficulties distributed towards the higher end of θ values. Individuals higher on the trait mind wandering continuum were therefore less likely to respond to probes using options 1 “on-task” and 2 “task-related thought”, and more likely to respond using other options. This pattern lends support for the practice of dichotomizing these responses into on-task (response options 1 and 2) and off-task (response options 3 through 8) categories (Kane et al., 2016, 2021b).

**Fig. 6:**
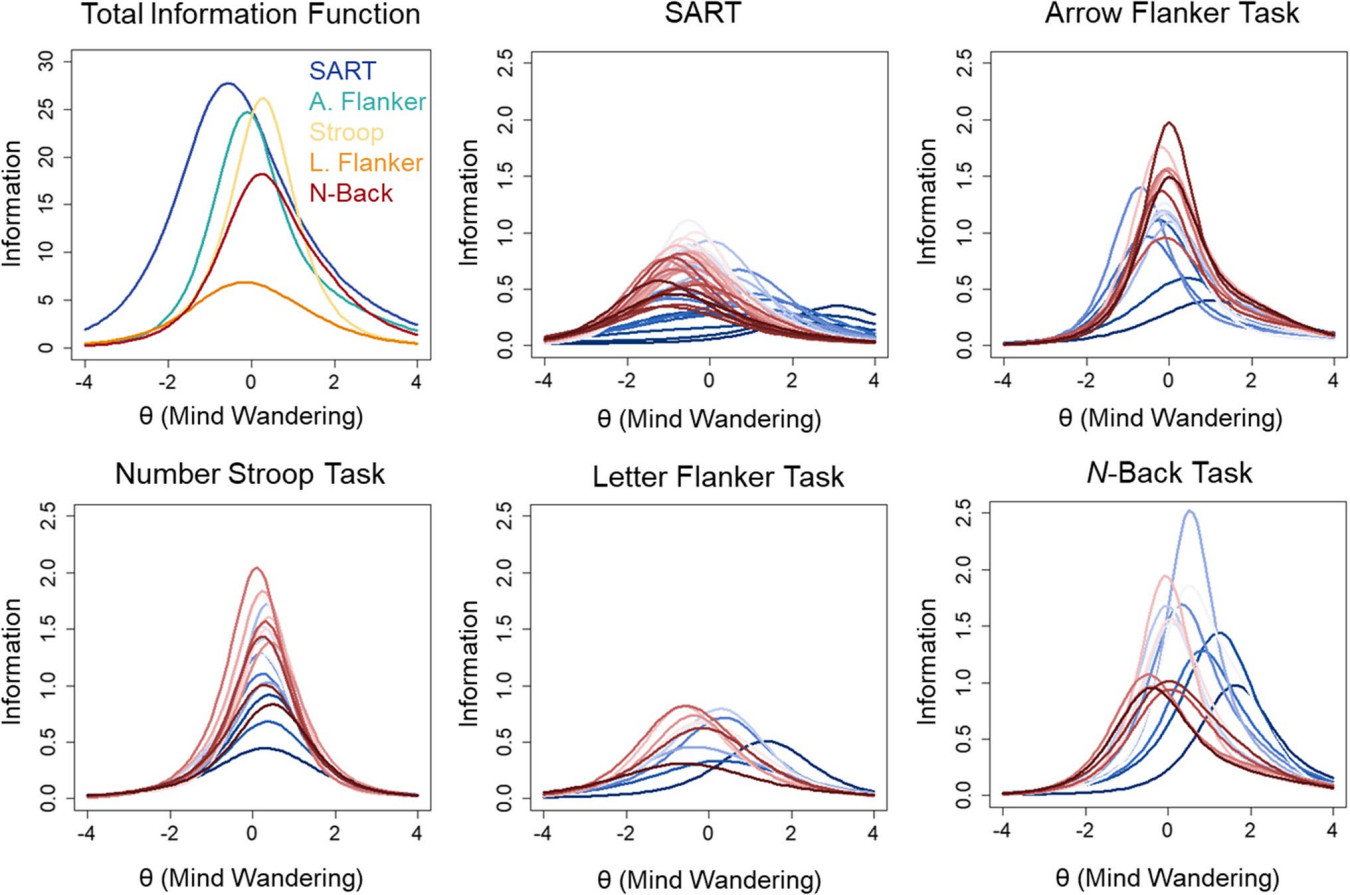
Total information for all five cognitive tasks from two-parameter nominal response models (2P NRM) of categorical probe ratings from Kane et al. (2016) is shown in the first panel (*top left*). The remaining panels show the item information curves with colors ranging from *dark blue* for early probes to *dark red* for later probes

Item information extended over a broad range of θ values across tasks (see Fig. 6). Earlier probes appeared to provide more total information than probes presented later in tasks. This distribution was confirmed by polynomial quadratic trends fit to the information values across sequential probes (see Supplementary Fig. 1). The trends were significant and explained 74% of the variance in the SART, *R*^2^ = 0.740, *F*(2,42) = 59.65, *p* < .001, 67% of the variance in the Arrow Flanker, *R*^2^ = 0.665, *F*(2,17) = 16.87, *p* < .001, 71% of the variance in the Stroop, *R*^2^ = 0.710, *F*(2,17) = 20.83, *p* < .001, 61% of the variance in the Letter Flanker, *R*^2^ = 0.606, *F*(2,9) = 6.92, *p* = .015. The N-Back was the exception to this pattern. Earlier N-Back probes provided more information than later probes: quadratic trends explained 54% of the variance, *R*^2^ = 0.539, *F*(2,12) = 7.03, *p* = .010.

**Fig. 7:**
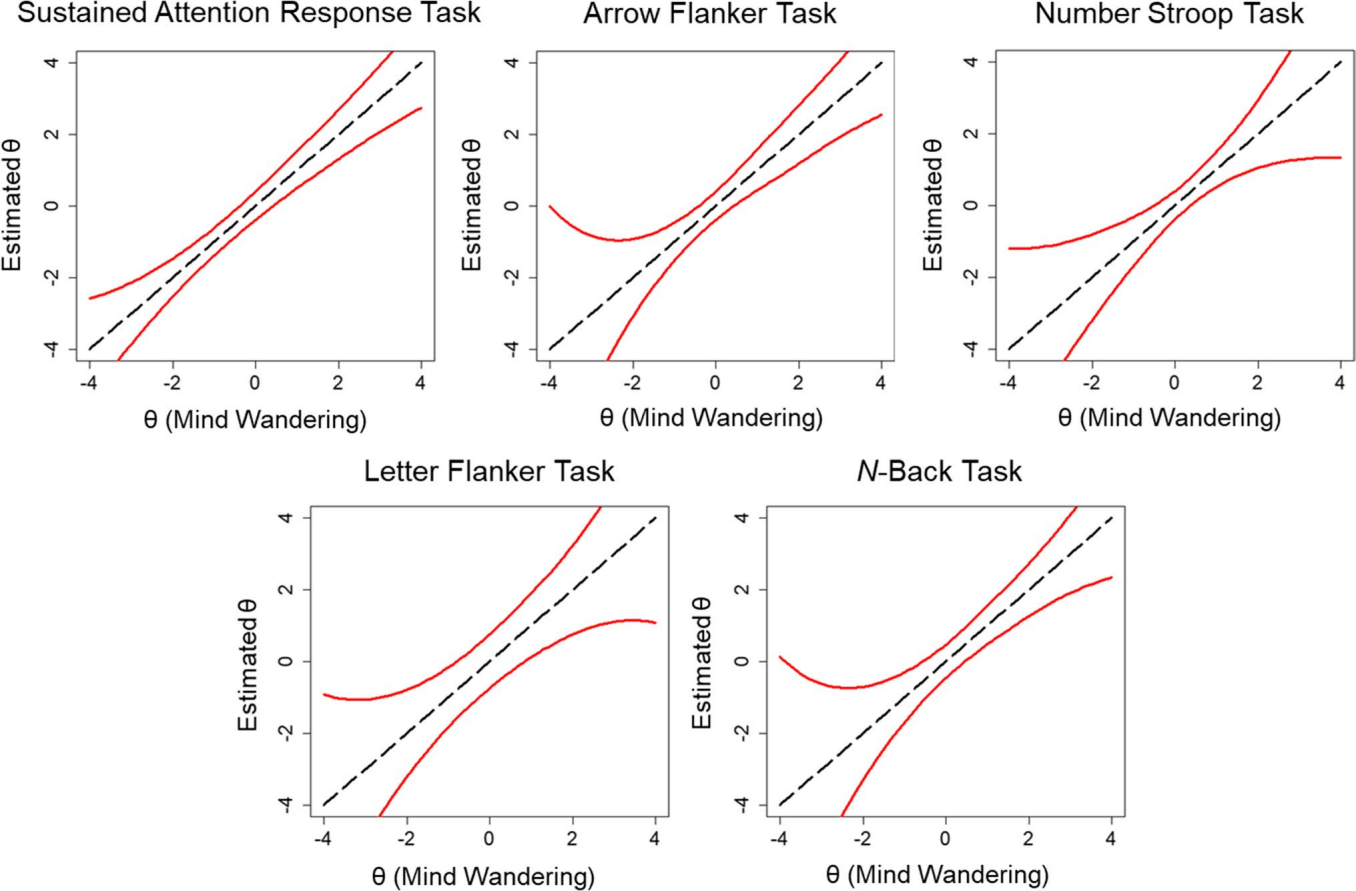
Measurement precision of estimates of trait mind wandering (θ) are shown for all five cognitive tasks from Kane et al. (2016) based on the total information from two-parameter nominal response models (2P NRM) of categorical probes. The *red line* depicts the 95% CI around θ estimates at all levels of θ

The total information was also greater for the SART (information = 105.47) relative to the Arrow Flanker (67.35), Stroop (60.39), Letter Flanker (26.00), and *N*-Back (55.72) tasks. Only 44.41% of the total information was contained within θ = – 1 to 1 for the SART, whereas 58.47% was contained within those bounds for the Arrow Flanker, 64.78% for the Stroop, 48.03% for the Letter Flanker, and 53.76% for the N-Back tasks. Importantly, response categories 1 “on-task”, 2 “task-unrelated thought”, 4 “current state”, and 6 “daydreams” provided most of the information about latent mind wandering: 86.18% of the total information for the SART, 83.20% for the Arrow Flanker, 84.00% for the Stroop, 82.44% for the Letter Flanker, and 83.98% for the N-Back tasks. Other response options provided less information about mind wandering tendency. Finally, based on the distribution of total item information, the 95% CIs around θ estimates demonstrated that θ estimates were relatively precise for values 2 standard deviations above and below the mean for all tasks (see Fig. 7). Estimates were more precise across a broader range of values for the SART than other tasks.

#### Two-parameter logistic model for dichotomized ratings

A 1PL and 2PL model were fit to the data from individuals who completed five cognitive tasks with embedded mind wandering probes. In line with studies utilizing categorical probe options to calculate the proportion of off-task mind wandering probes (e.g., Kane et al., 2016, 2021b), we dichotomized the eight categorical response options into on- and off-task categories by classifying ratings of 1 “on-task” and 2 “task-related thought” into on-task episodes and all other ratings into off-task episodes. The 2PL model fit the data significantly better fit than the 1PL model for all cognitive tasks except for the Letter Flanker task (see Table 3). The results of the 2PL are described here and item parameters are provided for all tasks in Supplementary Materials (Supplementary Tables 10 through 14).

**Table 3:** Model fit of the one- and two-parameter logistic model

Task	Model	AIC	BIC	Log-Lik.	χ^2^	*df*	*p*-value
SART	1PL	25364.5	25560.7	– 12636.2			
	2PL	25338.7	25722.6	– 12579.3	114	44	< .001
Arrow Flanker	1PL	10331.7	10419.3	– 5144.8			
	2PL	10326.1	10493.0	– 5123.1	43.5	19	.001
Num. Stroop	1PL	10315.6	10403.1	– 5136.8			
	2PL	10306.9	10473.7	– 5113.5	46.7	19	< .001
Letter Flanker	1PL	6485.0	6538.8	– 3229.5			
	2PL	6495.7	6595.0	– 3223.9	11.3	11	.420
*N*-Back	1PL	6903.8	6969.9	– 3435.9			
	2PL	6899.1	7023.1	– 3419.6	32.7	14	.003

Model comparisons of 1PL and 2PL models for five cognitive tasks from Kane et al. (2016) are provided alongside the Akaike information criterion (AIC), Bayesian information criterion (BIC), and log-likelihood of each model. The χ2 log-likelihood ratio test between models is given with degrees of freedom (*df*) and accompanying *p* values

**Fig. 8:**
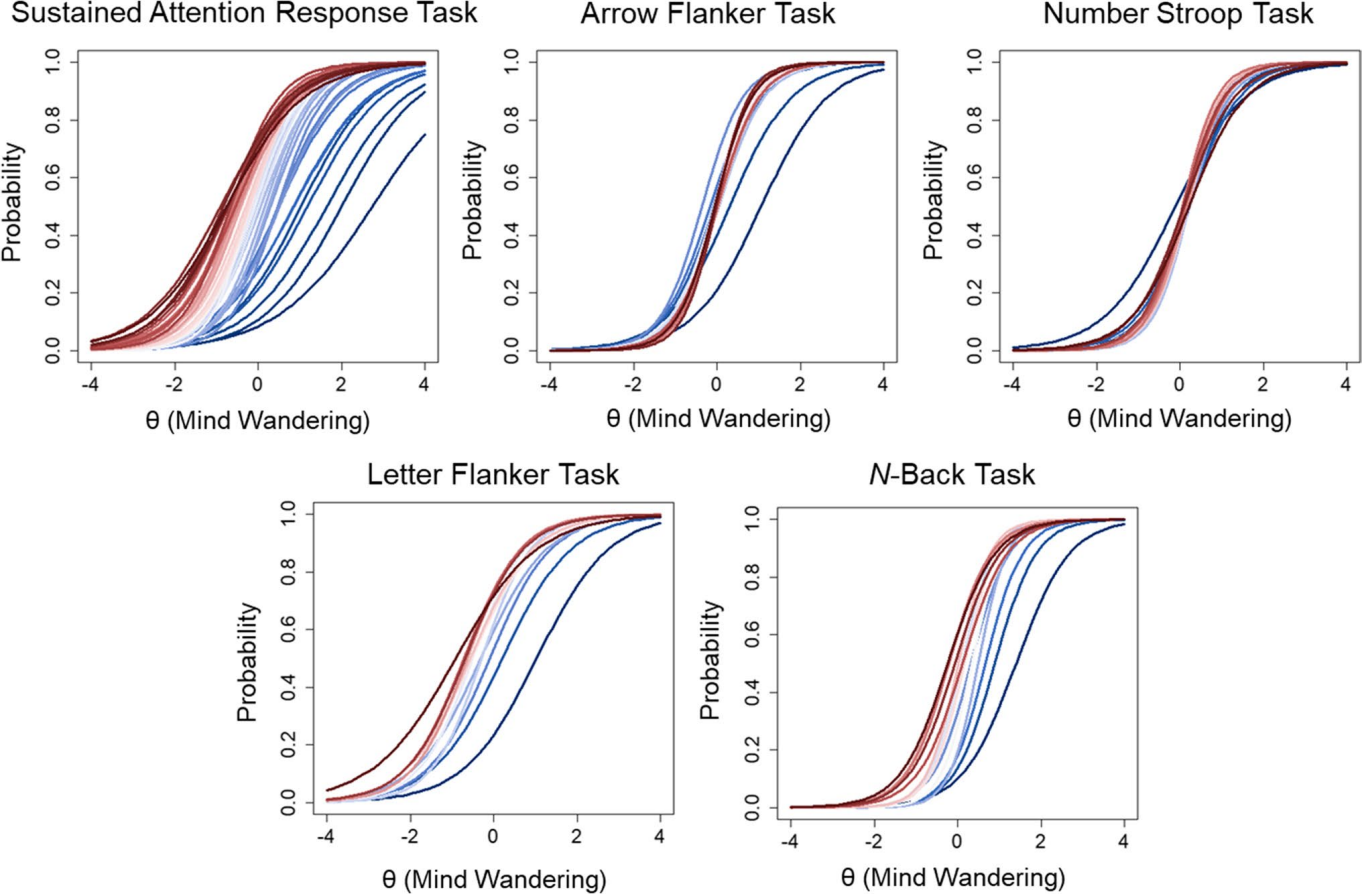
Item characteristic curves are shown for all five cognitive tasks based on two-parameter logistic (2PL) models of dichotomized probe ratings from Kane et al. (2016). Each *curve* represents the probability of an individual endorsing an “off-task” response at all levels of trait mind wandering (θ). Curves range in colors from *dark blue* for early probes to *dark red* for later probes

Item discrimination parameters (*a*_*i*_) for SART (range, 0.87–2.21), Arrow Flanker (range, 1.25–2.53), Stroop (range, 1.18–2.38), Letter Flanker (range, 1.01–1.67), and *N*-Back (range, 1.57–2.88), were high across mind wandering probes. Item difficulty parameters (*b*_*i*_) were distributed across a narrower range of θ values for SART (range, – 0.90 to 2.74), Arrow Flanker (range, – 0.36 to 1.06), Stroop (range, – 0.11 to 0.23), Letter Flanker (range, –0.91 to 1.03), and *N*-Back tasks (range, – 0.24 to 1.40). Difficulty parameters for the SART were distributed across a larger range of values than other tasks. This was evident when examining the item characteristic curves for all probes based on the 1PL model parameters for each task (see Fig. 8). Across tasks, early probes (Fig. 8, blue lines) appeared to have higher difficulty values than later probes (red lines).

**Fig. 9:**
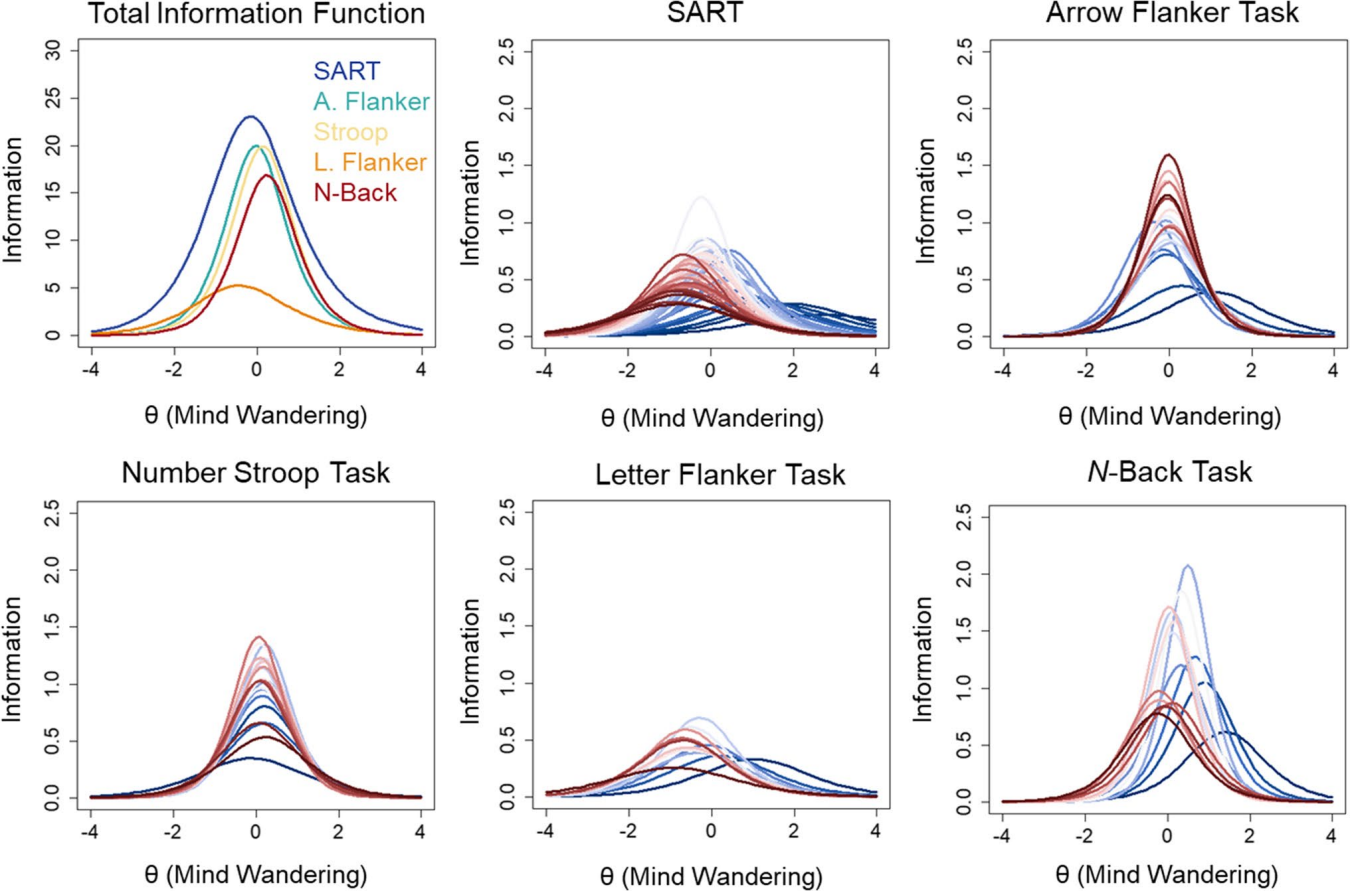
Total information for all five cognitive tasks based on two-parameter logistic (2PL) models of dichotomized probe ratings from Kane et al. (2016) is shown in the first panel (*top left*). The remaining panels show the item information curves with colors ranging from *dark blue* for early probes to *dark red* for later probes

**Fig. 10:**
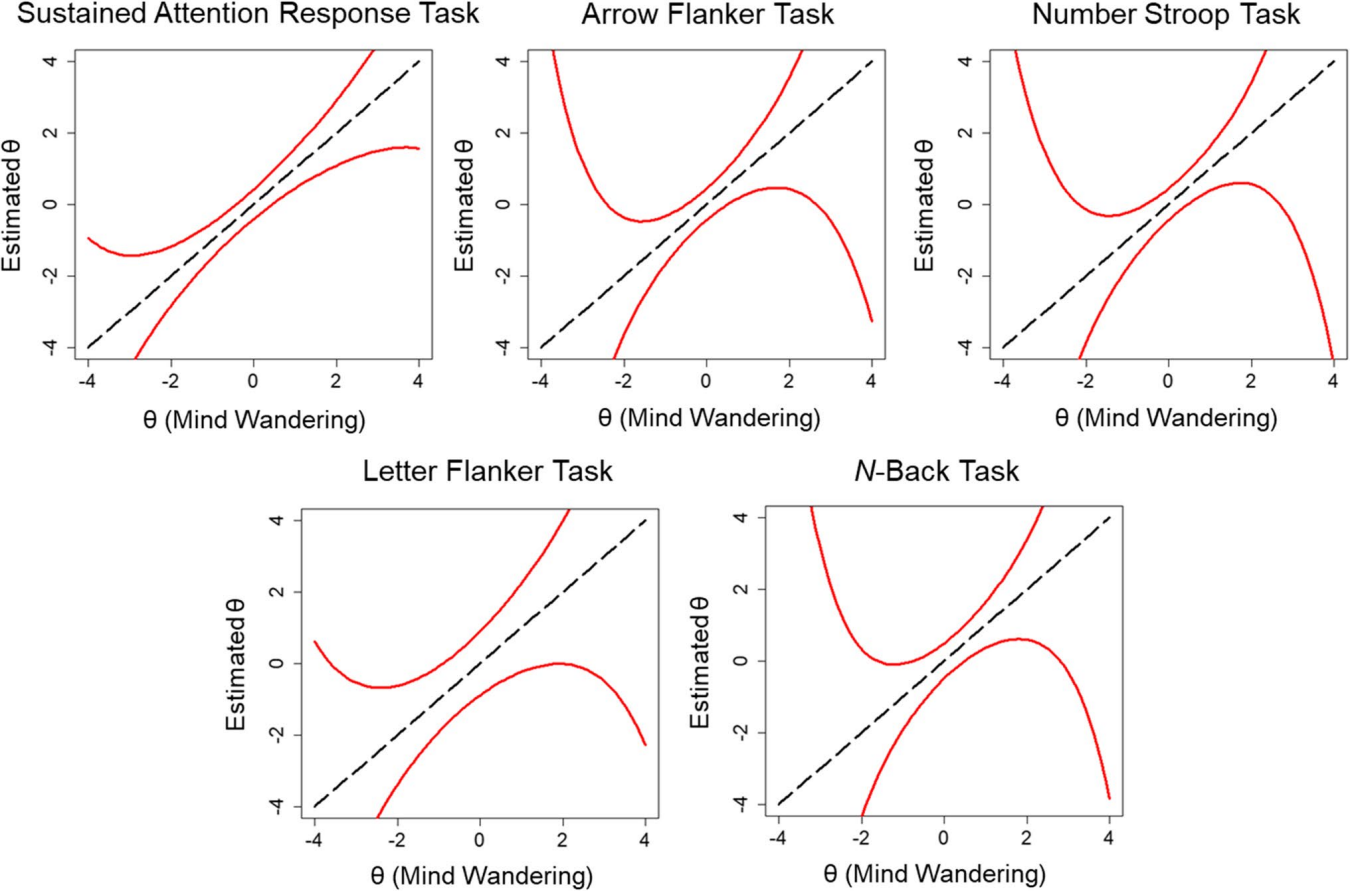
Measurement precision of estimates of trait mind wandering (θ) are shown for all five cognitive tasks from Kane et al. (2016) based on the total information from two-parameter logistic (2PL) models of dichotomized probes. The *red line* depicts the 95% CI around θ estimates at all levels of θ

Item information tended to be restricted to a narrow range of θ values across tasks (see Fig. 9). Earlier probes provided more information towards the higher end of the trait continuum than later probes. The total test information was greater for the SART (information = 66.53) relative to the Arrow Flanker (39.75), Stroop (39.57), Letter Flanker (16.19), and *N*-Back (33.16) tasks. This represented an overall reduction in information when categorical ratings were further dichotomized into on-task and off-task categories for the SART (36.92% reduction), Arrow Flanker (40.98%), Stroop (34.48%), Letter Flanker (37.73%), and *N*-Back (32.29%) tasks. Only 58.82% of the total information was contained within θ = – 1 to 1 for the SART, whereas 75.45% was contained within those bounds for the Arrow Flanker, 75.29% for the Stroop, 53.97% for the Letter Flanker, and 74.41% for the *N*-Back tasks. The 95% CIs around θ estimates demonstrated that θ estimates were imprecise for values 1 standard deviation above and below the mean for all tasks (see Fig. 10). Estimates were more precise across a broader range of values for the SART than other tasks. However, measurement precision was generally poor for θs across the lower and higher range of the trait continuum.

## Discussion

We examined the psychometric properties of experience sampling mind wandering ratings using methods from Item Response Theory (IRT) through secondary analyses of three independent data sets. IRT modeling demonstrated that mind wandering probes delivered earlier in ordinal position in tasks had lower discrimination parameter values than later probes, indicating that earlier probes provided less total information than later probes. Information from earlier probes also tended to be distributed towards the higher range of the trait continuum. Probe ratings made with continuous rating options also provided more information about individuals’ latent mind wandering tendency – across a broader range of the trait continuum – than ratings dichotomized into on-task and off-task categories. This was evident from test information provided by intermediate positions of the continuous rating scale and from reductions in information when continuous ratings were dichotomized. Specifically, ratings made on intermediate positions of the continuous scale provide substantial additional information about trait mind wandering, most notably in differentiating individuals at the lower and higher ends of the trait continuum. Together, these findings support the use of experience sampling probes to measure mind wandering and suggest several avenues for increasing the measurement precision of task-embedded probes.

Our primary goal was to evaluate the measurement precision of various mind wandering probe procedures using IRT methods. We found that item information was spread across the range of trait estimates according to when probes were presented ordinally in time. Probes presented earlier in ordinal position had higher difficulty but lower discrimination parameter values than later probes, indicating that they provided less total information but provided more information about individuals who had greater trait mind wandering. This aligns with time-ordered increases in mind wandering reliably observed across studies (i.e., Brosowsky et al., 2023; Krimsky et al., 2017; Thomson et al., 2014; Zanesco et al., 2020, 2024). Indeed, only those individuals who are highly prone to mind wandering would be expected to report being off task at the start of their engagement, whereas even highly focused individuals are likely to report episodes of mind wandering at later probes as their focus wanes over time. Responses to earlier probes also provided less overall information and were less indicative of individuals’ mind wandering propensity. This suggests that tasks shorter in duration may provide less reliable estimates of mind wandering than longer tasks that provide more opportunities for individuals’ attention to lapse and wander off-task.

A large-sample meta-analysis of experience sampling studies of mind wandering found that studies typically present around 30 total mind wandering probes on average at a frequency of roughly one probe per minute during cognitive tasks or activities (Zanesco et al., 2024). Tasks with shorter durations tend to have fewer total probes. Our findings provide some indication that longer tasks provide more information about individuals’ mind wandering tendency because greater numbers of total probes increase measurement precision and longer tasks provide individuals of differing propensity more opportunities to mind wander. However, presenting probes too frequently also reduces overall rates of probe-caught mind wandering (Schubert et al., 2020; Zanesco et al., 2024). Longer tasks can administer an adequate number of probes while maintaining an acceptable probe presentation rate. Tasks that facilitate the rate of growth in mind wandering over time may lead to better assessment of mind wandering by providing greater measurement information that varies across a broader range of the trait continuum. Despite these considerations, Welhaf et al. (2023) provided some evidence that as few as eight probes can provide reliable estimates of individuals’ mind wandering. Future studies ought to examine the relative benefit of task length and probe presentation rate to the measurement precision of probes by directly manipulating these factors (cf. Welhaf et al., 2023).

We also showed that continuous, polytomous probe ratings provide improved measurement precision of latent mind wandering tendency relative to when ratings are dichotomized into on-task and off-task states, regardless of the kind of task. In both studies by Zanesco et al. (2020) and Goller et al. (2020), intermediate ratings contributed substantial information about latent mind wandering tendency across a broader range of the trait continuum beyond that provided by ratings of 1 “on-task” and 6 “off-task” alone. Furthermore, when continuous ratings were dichotomized into on-task and off-task states, probes provided information in a narrower range of the latent trait continuum. This pattern was also evident in the content-based, categorical ratings made in the study by Kane et al. (2016), in which dichotomized probe ratings provided less total information. Overall, these findings provide some indication that dichotomous response options (or dichotomizing continuous or multiple categorical options into on-task or off-task categories) provide less measurement information than other methods, despite these approaches being the most common response options used in experience sampling studies of mind wandering (Weinstein, 2018).

The measurement precision of mind wandering probes is consequential because poor precision at the ends of the trait continuum implies that probes are not able to accurately assess trait mind wandering in highly task-focused individuals who experience little mind wandering or in those more prone to being off-task. We nevertheless found that continuous probe ratings provide better measurement of individuals’ trait mind wandering at the lower and higher ends of the latent trait continuum than dichotomized probes. This is perhaps intuitive because ratings ranging along gradations from “on-task” to “off-task” provide more opportunities for focused or unfocused individuals (e.g., individuals rating their focus as “moderately off-task” on average) to distinguish themselves from more highly focused or unfocused individuals (e.g., rating their focus as “completely off-task”). In contrast, ratings dichotomized into on-task and off-task categories collapse across this potential variability, eliminating subtler distinctions between individuals. Most of the measurement information in data sets using dichotomous probes were clustered around the average (θ = 0). As such, dichotomous probe methods may be less effective at measuring mind wandering in populations at the lower and higher ends of the latent trait continuum.

The phrasing of questions accompanying probes might have to be specially adapted to better target populations that experience little or excessive levels of mind wandering. For example, Mowlem et al. (2019a) developed their self-report questionnaire with the aim of assessing mind wandering in individuals prone to excessive levels of mind wandering in daily life and adapted the wording of questions accordingly. Researchers ought to consider the propensity for mind wandering in the population they are sampling from when designing their study. It might be necessary to adapt experience sampling procedures to better target samples of individuals more prone to experiencing mind wandering, such as individuals with ADHD (Mowlem et al., 2019a, b), or in those who are highly task-focused and infrequently report episodes of mind wandering during directed cognitive tasks, such as proficient mindfulness meditation practitioners (e.g., Zanesco et al., 2016). Reliability of probe ratings can also be improved by clearly identifying response category labels and communicating instructions to participants. As noted by Seli et al. (2018c), it is valuable for researchers to directly report how they conceptualized and operationalized mind wandering in their studies as well as how they explained these concepts to their participants.

IRT modeling of content-based categorical ratings from the study by Kane et al. (2016) also suggested good levels of measurement precision from these probes. The eight categorical response options provided adequate levels of information across a broad range of the trait continuum. Interestingly, these analyses also provide further justification for considering task-related thoughts (i.e., task-related interference) as contributing to the task-focused dimension of the construct continuum. Individuals with lower trait tendency to mind wander (i.e., lower θ) were more likely to respond to probes using options “on-task” and “task-unrelated thought”, and less likely to respond using other options. This lends support for the practice of grouping these two response options into an “on-task” category. In contrast, “daydreams” and “thoughts about one’s current state” were response options that provided the most information about the higher end of the trait continuum, as those options were primarily used by individuals with greater trait mind wandering. Together, these four response options provided most of the information from among the eight possible responses.

In the future, researchers might consider reducing the number of response options available to participants because several options provided little information about trait mind wandering (e.g., thoughts about the “environment” and “other” experiences). Alternatively, these response options might provide little information about individuals’ attentional state in the assessment context of a quiet laboratory but may be more meaningful when mind wandering is assessed in situations with more pronounced environmental interference. One recommendation for researchers considering content-based categorical response options is to avoid dichotomizing ratings into on- and off-task categories if possible. Instead, researchers might utilize multinomial or multivariate analytic approaches, such as multinomial logistic regression or sequence analysis of categorical time series data (Zanesco, 2020), so that the rich categorical information about individuals’ mental states can be retained. After all, prioritizing collection of data about the many kinds of thoughts and experiences occurring during task performance seems at odds with discounting this information by dichotomizing responses. Kane et al. (2021b) note that content-based categorical probes have another important advantage because they may be less susceptible to confabulation, guessing, or other biases, because they require respondents to commit to a specific experience. This might help encourage careful, attentive response relative to other probe rating options.

It is also important that researchers work to empirically determine the optimal number of rating options for their studies (Nunnally, 1967; Linacre, 2002). There may be diminishing returns to increasing the number of rating options because individuals may not be able to reliably discriminate between too many categories of responses. Providing too many response options might also lead to a frustrating participant experience. On the other hand, it is also critical that probes provide enough rating options so that meaningful variation in responses are maintained. Future studies using IRT might adjudicate between these considerations by evaluating whether rating categories demonstrate disordered difficulty thresholds. Specifically, the generalized partial credit model freely estimates the probability of responding to specific categories and can be used to evaluate whether continuous response categories demonstrate proper ordering in their difficulty thresholds along the trait continuum. In contrast, the graded response model cannot identify disordered thresholds because categories are ordered by design. Disordered thresholds can be indicative of response categories that are infrequently used by respondents (Adams et al., 2012), which may identify response categories that can be revised or removed from subsequent applications to further optimize the rating scale.

Across studies, probes administered during the SART appeared to be better at assessing mind wandering across a broader range of the trait continuum than other tasks, either because more overall probes were incorporated into the task or because task features better distributed information across the trait continuum. This latter possibility is generally consistent with the unexamined sentiment in the literature – the SART being widely utilized in studies of mind wandering – that it is an optimal task for measuring mind wandering, given its duration, facilitation of attentional disengagement and boredom, and proposed dependence on sustained attention (Robertson et al., 1997). It will be valuable for future studies to employ IRT methods to directly evaluate which cognitive tasks are best for measuring mind wandering in the task performance context. Indeed, several task paradigms have been specifically developed with the aim to better assess mind wandering and related constructs (i.e., Anderson et al., 2021; Esterman et al., 2013; Laflamme et al., 2018; Levinson et al., 2014), but the psychometric properties of embedded mind wandering probes could be empirically evaluated in these tasks.

Some evidence suggests that different cognitive tasks assess mind wandering equally well. For example, mind wandering scores calculated from different tasks tend to correlate similarly with other individual differences measures of attentional control (e.g., Kane et al. 2016, 2021b; Robison et al., 2020; Welhaf et al., 2020, 2023), and latent variable studies have found that mind wandering scores from different tasks tend to load on a single factor (e.g., Kane et al., 2016; Kane & McVay, 2012; Robison et al., 2020; Unsworth et al., 2020). Yet, correlations between individuals’ mind wandering scores from different tasks also suggest appreciable variability in rates of mind wandering across contexts or tasks (Kane et al. 2016, 2021b; Kane & McVay, 2012; Unsworth et al., 2020; Welhaf et al., 2020; Zanesco, 2020), and factors such as task demand, length, and probe presentation rate have been shown to influence rates of mind wandering (Krimsky et al., 2017; Schubert et al., 2020; Seli et al., 2013; Thomson et al., 2014; Zanesco et al., 2024). Indeed, a recent individual participant data meta-analysis demonstrated considerable heterogeneity in rates of mind wandering across study samples and different task performance contexts (Zanesco et al., 2024). While this meta-analysis did not identify systematic and reliable differences in rates of mind wandering between specific tasks, it is nevertheless possible that some tasks might be better suited for measuring mind wandering than others.

Probe ratings made with continuous, polytomous response options assume that task-focus and mind wandering exist on ends of a continuum, despite few attempts to conceptually specify the mental states existing along this gradation (Tay & Jebb, 2018). Alternatively, it is possible that the distribution of responses reflects a dichotomous mental state and gradations in continuous ratings only reflect measurement error. Statistical methods exist that aim to empirically adjudicate whether individuals can meaningfully utilize continuous response options or whether variation in individuals’ continuous responses result from measurement error. Specifically, taxometrics is a family of statistical and graphical techniques designed to evaluate whether a construct of interest is dichotomous or continuous in nature (Meehl, 1995). These methods may therefore be useful in resolving ambiguity regarding whether individuals’ use of continuous probe response options reflects gradations in the experience of mind wandering. Signal detection approaches have also been useful for distinguishing lapse rates – as might result from “all-or-nothing” episodes of mind wandering – from shifts in criterion or sensitivity (e.g., Gyles et al., 2023; McCarley & Yamani, 2021; Román-Caballero et al., 2023). It will also be valuable to explore whether event-related potential responses and electrophysiological brain states in cognitive tasks form bimodal distributions accompanying on- or off-task reports, or, alternatively, whether they vary continuously with polytomous mind wandering ratings.

Although the present study provides empirical support for the use of continuous probe rating procedures, it does not address other potential threats to the construct validity of these methods. Kane et al. (2021b) compared the construct validity of continuous and dichotomized mind wandering ratings and concluded that continuous ratings showed potential threats to their construct validity and had no incremental benefit beyond dichotomized, categorical ratings. Specifically, individuals were less confident overall when reporting they were off-task using continuous ratings than dichotomized, categorical content-based judgements (Kane et al., 2021b). Some of these individuals had the least confidence in their most extreme off-task ratings, whereas others were least confident in their mixed ratings halfway between on-task and off-task. This suggests variance in how people *use* continuous rating options to judge their mind wandering, raising concern about the construct validity of these probes. Continuous ratings also appeared to be more biased by ongoing performance than other probe types, perhaps because individuals were less confident in their ratings and were using objective cues about their performance to guide judgements about their current mental state. Kane et al. (2021b) also found that continuous ratings tended to show no incremental benefit beyond dichotomized ratings based on the magnitude of individual differences correlations. These potential threats to validity will have to be weighed alongside potential measurement benefits when researchers decide on which methods to use in their studies.

The requirements of IRT approaches for larger sample sizes are a particular limitation to the psychometric evaluation of instruments or tests in many contexts. Conventional recommendations and simulation studies suggest that obtaining reliable IRT estimates requires sample sizes ranging from 500 to 2000 respondents (see Finch & French, 2019, for discussion). However, the complexity of the IRT model and other factors can also influence recommendations for acceptable sample size. While we utilized several large-sample data sets in our present investigation, the IRT analyses presented here should be considered tentative until additional larger samples can corroborate the present results. Another limitation of our work is that IRT analyses assumed that sequential probes were separate items or indicators of a single test of mind wandering propensity in the same manner that items of a questionnaire might assess a single construct. However, unlike a questionnaire instrument, probes presented the same question at each sequential sample and considerable time passed between probes. We only examined probes with respect to their ordinal position and did not incorporate information about the exact chronological timing of probes into our IRT analyses. Although these assumptions are made for other kinds of tests or questionnaire instruments as well, test items can typically be randomized to reduce the influence of presentation order and timing on IRT estimates. This is not possible when the same probe question is presented throughout the task, and researchers are often specifically interested in the influence of presentation order and timing on rates of mind wandering (e.g., Zanesco et al., 2024).

As is the case in many fields of psychological research (Flake & Fried, 2020; Shaw et al., 2020), mind wandering probe questions and response options are largely utilized ad hoc by different research groups with little systematic effort to confirm their reliability and validity or maximize their measurement precision. It is critical for our psychological understanding of mind wandering that future research continues to focus on issues of measurement. The present psychometric investigation contributes to this effort by providing some guidance on the choice of probe rating methods for the assessment of trait mind wandering tendencies. We hope this information will guide researchers in choosing probe rating methods that maximize the measurement precision of their assessment procedures, as well as motivate future research on the measurement of mind wandering using experience sampling probes. Research investigating the optimal methods for assessing mind wandering in specific populations or tasks contributes both to practical solutions for designing better measurement instruments and our theoretical understanding of the introspective ability of individuals to notice and reflect on their wandering mind.

### Supplementary Information

Below is the link to the electronic supplementary material.

Supplementary file1 (DOCX 479 KB)

## Data Availability

The conclusions of this study are based on secondary analyses of data that are available on the Open Science Framework for studies by Zanesco et al. (2020; https://osf.io/rf9jh/), Goller et al. (2020; https://osf.io/shxb6/), and Kane et al. (2016; https://osf.io/guhw7/). Curated data files used in this study are also provided online in the OSF repository and can be found at: https://osf.io/3zma2/

## Code availability

Analysis scripts can be found at: https://osf.io/3zma2/
