# Supplementary material for: Measuring mind wandering with experience sampling during task performance: An item response theory investigation
Source: Behav Res Methods. 2024 Jul 25;56(7):7707–27. doi: 10.3758/s13428-024-02446-9 (PMC11362314; doi:10.3758/s13428-024-02446-9)
Supplement: Supplementary file 1 — Supplementary file1 (DOCX 479 KB) [file 13428_2024_2446_MOESM1_ESM.docx]

**Supplemental Results**

**Two-parameter logistic** **model for dichotomized ratings of Zanesco et al. (2020).** In supplemental analyses, we dichotomized the continuous ratings into on- and off-task categories by classifying ratings of 1 into on-task episodes and ratings of 2, 3, 4, 5, and 6 into off-task episodes. A two-parameter logistic (2PL) model was fit to the dichotomous ratings and item discrimination and difficulty parameters are provided in Supplementary Table 15. Item difficulty parameters (*b_i_*) ranged from -0.02 to 1.17, whereas item discrimination parameters (*a_i_*) ranged from 1.73 to 5.00. Early probes had lower discrimination and higher difficulty values than later probes. Supplementary Figure 2 depicts the item characteristic curves for all probes based on the 2PL model parameters. The item characteristic curves of early probes (Supplementary Figure 2A, blue lines) were distributed towards the higher end of θ values compared to later probes (red lines).

Item information was high in a narrow range of θ values (see Supplementary Figure 2B), with early probes providing more information at the higher end of the latent continuum. The total test information from all 28 mind wandering probes (total information = 95.44) was also narrowly distributed around θ = 0 and is shown in Supplementary Figure 2C. This represents a 48.68% reduction in information relative to the results of the two-parameter graded response model using continuous ratings. 78.88% of the total information from the 2PL model was contained within θ = -1 to θ = 1. This represents a shift in the distribution of information towards the center of the trait continuum (i.e., θ = 0), relative to when ratings > 3 were dichotomized into the off-task category. However, the total information did not differ relative to when ratings > 3 were dichotomized. Supplementary Figure 2D depicts the 95% CIs around θ estimates at all levels of θ. Measurement was less precise 1, 2, or 3 standard deviations above or below the mean. There was a loss in information from the lower and higher range of the trait continuum.

**Two-parameter logistic** **model for dichotomized ratings of Goller et al. (2020).** We also dichotomized the continuous ratings into on- and off-task categories by classifying ratings of 1 into on-task episodes and ratings of 2, 3, 4, and 5 into off-task episodes. A two-parameter logistic (2PL) model was fit to the data and item discrimination and difficulty parameters are provided in total in Supplementary Table 16. Item difficulty parameters (*b_i_*) ranged from -1.10 to 0.12, and item discrimination parameters (*a_i_*) ranged from 1.08 to 3.14. Early probes had lower discrimination and higher difficulty values than later probes. Supplementary Figure 3A depicts the item characteristic curves for all probes based on the 2PL model parameters. The item characteristic curves of early probes (Supplementary Figure 3A, blue lines) were distributed towards the higher end of θ values.

Item information was high in a narrow range of θ values (see Supplementary Figure 3B), with earlier probes (blue lines) being more distributed towards the higher end of the trait continuum. The total test information from all 38 mind wandering probes (total information = 83.54) is shown in Supplementary Figure 3C and was more narrowly distributed around the mean θ. 54.06% of total information from the 2PL model was contained within θ = -1 to θ = 1. Supplementary Figure 3D depicts the 95% CIs around θ estimates at all levels of θ. Measurement was more imprecise at differentiating individuals 1, 2, or 3 standard deviations above or below the mean. Information was therefore lost from the lower and higher range of the trait continuum when continuous ratings were dichotomized into on-task and off-task states.

| Supplementary Table 1: 2P GRM parameter estimates for Zanesco et al. (2020) | | | | | | | | |
| --- | --- | --- | --- | --- | --- | --- | --- | --- |
| Probe Num. | *a* | *b_1_* | *b_2_* | *b_3_* | *b_4_* | *b_5_* | Information | % Total Information |
| Probe 1 | 1.96 | 1.34 | 1.96 | 2.46 | 2.77 | 3.11 | 4.16 | 2.24 |
| Probe 2 | 1.50 | 1.03 | 1.80 | 2.49 | 2.71 | 3.07 | 2.98 | 1.60 |
| Probe 3 | 1.91 | 0.74 | 1.45 | 1.99 | 2.42 | 2.59 | 4.06 | 2.18 |
| Probe 4 | 2.49 | 1.25 | 1.75 | 2.13 | 2.52 | 2.75 | 5.51 | 2.96 |
| Probe 5 | 1.81 | 0.36 | 1.05 | 1.61 | 1.90 | 2.24 | 3.79 | 2.04 |
| Probe 6 | 1.80 | 0.42 | 1.04 | 1.64 | 2.12 | 2.55 | 4.03 | 2.17 |
| Probe 7 | 2.16 | 0.64 | 1.32 | 1.79 | 2.09 | 2.36 | 4.74 | 2.55 |
| Probe 8 | 2.23 | 0.66 | 1.19 | 1.65 | 1.98 | 2.21 | 4.72 | 2.54 |
| Probe 9 | 2.17 | 0.36 | 1.02 | 1.50 | 1.80 | 2.01 | 4.66 | 2.51 |
| Probe 10 | 3.08 | 0.94 | 1.44 | 1.77 | 1.98 | 2.13 | 6.69 | 3.60 |
| Probe 11 | 2.11 | 0.23 | 0.82 | 1.24 | 1.63 | 2.02 | 4.69 | 2.52 |
| Probe 12 | 3.14 | 0.49 | 1.01 | 1.39 | 1.68 | 1.99 | 7.81 | 4.20 |
| Probe 13 | 4.06 | 0.78 | 1.21 | 1.60 | 1.78 | 2.00 | 10.31 | 5.54 |
| Probe 14 | 4.58 | 0.80 | 1.21 | 1.53 | 1.71 | 1.93 | 11.95 | 6.43 |
| Probe 15 | 4.08 | 0.84 | 1.24 | 1.55 | 1.72 | 1.87 | 9.50 | 5.11 |
| Probe 16 | 4.20 | 0.83 | 1.23 | 1.59 | 1.85 | 1.96 | 10.42 | 5.60 |
| Probe 17 | 2.60 | 0.42 | 1.03 | 1.51 | 1.78 | 2.01 | 5.99 | 3.22 |
| Probe 18 | 2.72 | 0.29 | 0.99 | 1.43 | 1.77 | 1.99 | 6.67 | 3.58 |
| Probe 19 | 4.21 | 0.82 | 1.23 | 1.53 | 1.79 | 2.05 | 11.08 | 5.96 |
| Probe 20 | 3.09 | 0.44 | 0.93 | 1.33 | 1.52 | 1.75 | 7.07 | 3.80 |
| Probe 21 | 2.55 | 0.19 | 0.71 | 1.13 | 1.47 | 1.69 | 5.69 | 3.06 |
| Probe 22 | 2.90 | 0.39 | 0.95 | 1.25 | 1.52 | 1.78 | 6.64 | 3.57 |
| Probe 23 | 3.55 | 0.61 | 1.14 | 1.40 | 1.69 | 1.86 | 8.46 | 4.55 |
| Probe 24 | 3.31 | 0.35 | 0.85 | 1.29 | 1.59 | 1.72 | 8.00 | 4.30 |
| Probe 25 | 3.76 | 0.58 | 1.01 | 1.37 | 1.61 | 1.81 | 9.27 | 4.98 |
| Probe 26 | 2.82 | 0.32 | 0.83 | 1.21 | 1.42 | 1.62 | 6.13 | 3.30 |
| Probe 27 | 2.62 | 0.29 | 0.84 | 1.22 | 1.51 | 1.73 | 5.79 | 3.11 |
| Probe 28 | 2.37 | 0.03 | 0.56 | 0.98 | 1.28 | 1.57 | 5.15 | 2.77 |

| Supplementary Table 2: 2PL parameter estimates for dichotomized ratings for Zanesco et al. (2020) | | | | |
| --- | --- | --- | --- | --- |
| Probe Num. | *a* | *b* | Information | % Total Information |
| Probe 1 | 1.77 | 2.55 | 1.77 | 1.83 |
| Probe 2 | 2.08 | 2.05 | 2.08 | 2.16 |
| Probe 3 | 1.95 | 1.92 | 1.95 | 2.02 |
| Probe 4 | 2.24 | 2.15 | 2.24 | 2.32 |
| Probe 5 | 2.09 | 1.42 | 2.09 | 2.17 |
| Probe 6 | 2.14 | 1.46 | 2.14 | 2.22 |
| Probe 7 | 2.78 | 1.57 | 2.78 | 2.88 |
| Probe 8 | 2.82 | 1.46 | 2.82 | 2.92 |
| Probe 9 | 2.74 | 1.30 | 2.74 | 2.85 |
| Probe 10 | 3.90 | 1.63 | 3.90 | 4.04 |
| Probe 11 | 2.05 | 1.17 | 2.05 | 2.13 |
| Probe 12 | 3.13 | 1.27 | 3.13 | 3.25 |
| Probe 13 | 4.94 | 1.46 | 4.94 | 5.13 |
| Probe 14 | 6.87 | 1.38 | 6.87 | 7.12 |
| Probe 15 | 5.93 | 1.40 | 5.92 | 6.15 |
| Probe 16 | 4.80 | 1.49 | 4.80 | 4.98 |
| Probe 17 | 2.68 | 1.42 | 2.68 | 2.78 |
| Probe 18 | 3.19 | 1.33 | 3.19 | 3.31 |
| Probe 19 | 5.17 | 1.43 | 5.17 | 5.37 |
| Probe 20 | 3.88 | 1.16 | 3.88 | 4.02 |
| Probe 21 | 2.99 | 0.97 | 2.99 | 3.11 |
| Probe 22 | 3.69 | 1.06 | 3.68 | 3.82 |
| Probe 23 | 4.18 | 1.27 | 4.18 | 4.34 |
| Probe 24 | 4.26 | 1.13 | 4.26 | 4.42 |
| Probe 25 | 4.92 | 1.24 | 4.91 | 5.10 |
| Probe 26 | 3.14 | 1.05 | 3.14 | 3.26 |
| Probe 27 | 3.11 | 1.05 | 3.11 | 3.23 |
| Probe 28 | 2.96 | 0.84 | 2.96 | 3.07 |

| Supplementary Table 3: 2P GRM parameter estimates for Goller et al. (2020) | | | | | | | | |
| --- | --- | --- | --- | --- | --- | --- | --- | --- |
| Probe Num. | *a* | *b_1_* | *b_2_* | *b_3_* | *b_4_* | Information | % Total Information |  |
| Probe 1 | 0.84 | 0.12 | 2.67 | 4.21 | 6.63 | 2.14 | 1.23 |  |
| Probe 2 | 0.96 | -0.38 | 1.38 | 3.19 | 4.92 | 2.43 | 1.40 |  |
| Probe 3 | 1.35 | -0.28 | 1.16 | 2.36 | 3.67 | 3.51 | 2.01 |  |
| Probe 4 | 1.14 | -0.15 | 1.19 | 2.79 | 3.67 | 2.66 | 1.53 |  |
| Probe 5 | 1.13 | -0.64 | 1.05 | 2.55 | 3.89 | 2.89 | 1.66 |  |
| Probe 6 | 1.44 | -0.34 | 0.88 | 1.99 | 3.32 | 3.73 | 2.14 |  |
| Probe 7 | 1.75 | -0.26 | 0.69 | 1.52 | 2.64 | 4.45 | 2.55 |  |
| Probe 8 | 1.55 | -0.55 | 0.55 | 1.66 | 2.21 | 3.60 | 2.06 |  |
| Probe 9 | 1.93 | -0.51 | 0.36 | 1.17 | 2.07 | 4.86 | 2.78 |  |
| Probe 10 | 1.79 | -0.31 | 0.53 | 1.29 | 2.15 | 4.22 | 2.42 |  |
| Probe 11 | 1.66 | -0.67 | 0.40 | 1.30 | 2.20 | 4.10 | 2.35 |  |
| Probe 12 | 1.82 | -0.48 | 0.46 | 1.17 | 1.90 | 4.26 | 2.44 |  |
| Probe 13 | 1.74 | -0.49 | 0.43 | 1.25 | 2.02 | 4.10 | 2.35 |  |
| Probe 14 | 1.81 | -0.69 | 0.27 | 1.16 | 1.80 | 4.34 | 2.49 |  |
| Probe 15 | 2.17 | -0.59 | 0.22 | 1.08 | 1.70 | 5.44 | 3.12 |  |
| Probe 16 | 2.19 | -0.44 | 0.32 | 1.01 | 1.73 | 5.39 | 3.09 |  |
| Probe 17 | 2.05 | -0.70 | 0.15 | 0.86 | 1.75 | 5.20 | 2.98 |  |
| Probe 18 | 2.54 | -0.61 | 0.13 | 0.90 | 1.49 | 6.61 | 3.79 |  |
| Probe 19 | 2.31 | -0.53 | 0.24 | 0.89 | 1.40 | 5.48 | 3.14 |  |
| Probe 20 | 2.23 | -0.56 | 0.17 | 0.86 | 1.50 | 5.38 | 3.08 |  |
| Probe 21 | 2.30 | -0.68 | 0.01 | 0.77 | 1.40 | 5.66 | 3.24 |  |
| Probe 22 | 2.40 | -0.61 | 0.08 | 0.76 | 1.31 | 5.80 | 3.32 |  |
| Probe 23 | 2.31 | -0.79 | -0.02 | 0.66 | 1.23 | 5.62 | 3.22 |  |
| Probe 24 | 2.50 | -0.82 | -0.01 | 0.55 | 1.24 | 6.40 | 3.67 |  |
| Probe 25 | 2.87 | -0.68 | 0.13 | 0.66 | 1.24 | 7.55 | 4.33 |  |
| Probe 26 | 2.31 | -0.96 | -0.04 | 0.64 | 1.19 | 5.78 | 3.32 |  |
| Probe 27 | 2.29 | -0.90 | -0.17 | 0.52 | 1.14 | 5.58 | 3.20 |  |
| Probe 28 | 2.19 | -0.86 | -0.03 | 0.58 | 1.16 | 5.18 | 2.97 |  |
| Probe 29 | 2.40 | -1.00 | -0.19 | 0.50 | 1.09 | 6.08 | 3.48 |  |
| Probe 30 | 2.06 | -0.97 | -0.12 | 0.53 | 1.09 | 4.77 | 2.73 |  |
| Probe 31 | 2.06 | -0.90 | -0.12 | 0.56 | 1.14 | 4.77 | 2.74 |  |
| Probe 32 | 2.29 | -1.08 | -0.19 | 0.45 | 0.95 | 5.53 | 3.17 |  |
| Probe 33 | 2.29 | -1.05 | -0.25 | 0.42 | 0.87 | 5.38 | 3.08 |  |
| Probe 34 | 2.70 | -0.88 | -0.19 | 0.41 | 0.97 | 6.82 | 3.91 |  |
| Probe 35 | 2.06 | -1.07 | -0.21 | 0.44 | 0.94 | 4.70 | 2.69 |  |
| Probe 36 | 1.77 | -1.13 | -0.27 | 0.56 | 1.16 | 4.02 | 2.31 |  |

| Supplementary Table 4: 2PL parameter estimates for dichotomized ratings for Goller et al. (2020) | | | | |
| --- | --- | --- | --- | --- |
| Probe Num. | *a* | *b* | Information | % Total Information |
| Probe 1 | 0.89 | 2.55 | 0.89 | 1.29 |
| Probe 2 | 1.01 | 1.30 | 1.01 | 1.46 |
| Probe 3 | 1.40 | 1.16 | 1.40 | 2.03 |
| Probe 4 | 1.33 | 1.05 | 1.33 | 1.93 |
| Probe 5 | 0.99 | 1.13 | 0.99 | 1.43 |
| Probe 6 | 1.36 | 0.90 | 1.36 | 1.97 |
| Probe 7 | 1.72 | 0.67 | 1.72 | 2.49 |
| Probe 8 | 1.44 | 0.55 | 1.44 | 2.08 |
| Probe 9 | 1.67 | 0.32 | 1.67 | 2.42 |
| Probe 10 | 1.53 | 0.56 | 1.53 | 2.21 |
| Probe 11 | 1.77 | 0.38 | 1.77 | 2.56 |
| Probe 12 | 1.56 | 0.49 | 1.56 | 2.26 |
| Probe 13 | 1.60 | 0.40 | 1.60 | 2.32 |
| Probe 14 | 1.66 | 0.24 | 1.66 | 2.41 |
| Probe 15 | 1.95 | 0.20 | 1.95 | 2.83 |
| Probe 16 | 2.02 | 0.28 | 2.02 | 2.93 |
| Probe 17 | 1.74 | 0.11 | 1.74 | 2.53 |
| Probe 18 | 2.57 | 0.07 | 2.57 | 3.73 |
| Probe 19 | 2.20 | 0.20 | 2.20 | 3.19 |
| Probe 20 | 2.20 | 0.11 | 2.20 | 3.18 |
| Probe 21 | 2.23 | -0.07 | 2.23 | 3.23 |
| Probe 22 | 2.63 | 0.02 | 2.63 | 3.81 |
| Probe 23 | 2.63 | -0.08 | 2.63 | 3.81 |
| Probe 24 | 2.66 | -0.08 | 2.66 | 3.85 |
| Probe 25 | 2.93 | 0.08 | 2.93 | 4.24 |
| Probe 26 | 2.37 | -0.09 | 2.37 | 3.44 |
| Probe 27 | 2.25 | -0.22 | 2.25 | 3.26 |
| Probe 28 | 2.02 | -0.10 | 2.02 | 2.93 |
| Probe 29 | 2.33 | -0.25 | 2.33 | 3.37 |
| Probe 30 | 2.03 | -0.18 | 2.03 | 2.94 |
| Probe 31 | 1.93 | -0.17 | 1.93 | 2.79 |
| Probe 32 | 2.14 | -0.27 | 2.13 | 3.09 |
| Probe 33 | 2.09 | -0.33 | 2.09 | 3.03 |
| Probe 34 | 2.54 | -0.26 | 2.54 | 3.68 |
| Probe 35 | 1.92 | -0.29 | 1.92 | 2.78 |
| Probe 36 | 1.73 | -0.34 | 1.73 | 2.50 |

| Supplementary Table 5: 2P NRM parameter estimates for multinomial ratings in the SART for Kane et al. (2016) | | | | | | | | | | | | | | | | | | | | | | | | | | | | | | | | |  |  |  |
| --- | --- | --- | --- | --- | --- | --- | --- | --- | --- | --- | --- | --- | --- | --- | --- | --- | --- | --- | --- | --- | --- | --- | --- | --- | --- | --- | --- | --- | --- | --- | --- | --- | --- | --- | --- |
| Probe Num. | | *a_1_* | | *a_2_* | *a_3_* | *a_4_* | | *a_5_* | | *a_6_* | | *a_7_* | | *a_8_* | | *c_1_* | | *c_2_* | | *c_3_* | | *c_4_* | | *c_5_* | | *c_6_* | | *c_7_* | | *c_8_* | |  |  |  |  |
| Probe 1 | | -0.40 | | -0.10 | -0.25 | 1.03 | | -0.66 | | 0.03 | | -0.30 | | 0.66 | | 2.80 | | 2.64 | | -0.64 | | -0.56 | | -2.13 | | 0.10 | | -0.94 | | -1.26 | |  |  |  |  |
| Probe 2 | | -0.66 | | -0.26 | 0.19 | 0.77 | | 0.15 | | -0.35 | | -0.34 | | 0.50 | | 2.07 | | 2.66 | | -0.51 | | -0.14 | | -1.12 | | -0.28 | | -1.20 | | -1.47 | |  |  |  |  |
| Probe 3 | | -0.83 | | -0.20 | -0.06 | 0.69 | | -0.39 | | 0.27 | | 0.06 | | 0.46 | | 1.78 | | 2.43 | | -0.18 | | 0.11 | | -1.56 | | -0.27 | | -0.89 | | -1.42 | |  |  |  |  |
| Probe 4 | | -1.13 | | -0.26 | 0.36 | 0.46 | | -0.07 | | 0.30 | | 0.00 | | 0.35 | | 1.41 | | 1.95 | | -0.37 | | 0.34 | | -1.07 | | -0.60 | | -0.44 | | -1.22 | |  |  |  |  |
| Probe 5 | | -1.14 | | -0.37 | -0.26 | 0.57 | | 0.31 | | 0.19 | | 0.25 | | 0.46 | | 1.57 | | 2.19 | | 0.01 | | 0.48 | | -1.06 | | -0.50 | | -1.22 | | -1.47 | |  |  |  |  |
| Probe 6 | | -1.06 | | -0.25 | 0.08 | 0.66 | | 0.32 | | 0.18 | | -0.21 | | 0.30 | | 1.17 | | 1.89 | | -0.02 | | 0.41 | | -1.14 | | -0.39 | | -0.95 | | -0.98 | |  |  |  |  |
| Probe 7 | | -1.11 | | -0.30 | 0.31 | 1.04 | | 0.39 | | 0.18 | | -0.74 | | 0.23 | | 1.24 | | 1.79 | | -0.36 | | -0.23 | | -0.74 | | -0.09 | | -0.63 | | -0.99 | |  |  |  |  |
| Probe 8 | | -1.24 | | -0.11 | 0.08 | 1.02 | | 0.15 | | 0.25 | | -0.29 | | 0.14 | | 0.70 | | 1.77 | | 0.05 | | 0.39 | | -0.62 | | -0.27 | | -1.05 | | -0.98 | |  |  |  |  |
| Probe 9 | | -1.44 | | -0.34 | -0.16 | 1.06 | | 0.39 | | -0.04 | | 0.22 | | 0.30 | | 0.79 | | 1.76 | | -0.14 | | 0.21 | | -0.47 | | 0.00 | | -0.80 | | -1.36 | |  |  |  |  |
| Probe 10 | | -1.64 | | -0.52 | -0.23 | 1.21 | | 0.38 | | 0.33 | | -0.14 | | 0.61 | | 0.89 | | 1.81 | | -0.30 | | 0.13 | | -0.70 | | -0.02 | | -0.63 | | -1.18 | |  |  |  |  |
| Probe 11 | | -1.46 | | -0.22 | -0.32 | 0.85 | | 0.72 | | 0.04 | | 0.10 | | 0.29 | | 0.75 | | 1.83 | | -0.22 | | 0.46 | | -1.00 | | 0.04 | | -0.87 | | -0.99 | |  |  |  |  |
| Probe 12 | | -1.76 | | -0.38 | -0.41 | 0.90 | | 0.72 | | 0.50 | | 0.15 | | 0.28 | | 0.70 | | 1.61 | | -0.15 | | 0.55 | | -0.97 | | 0.02 | | -0.74 | | -1.02 | |  |  |  |  |
| Probe 13 | | -1.67 | | -0.42 | 0.21 | 0.84 | | 0.46 | | 0.12 | | 0.21 | | 0.26 | | 0.70 | | 1.44 | | -0.53 | | 0.41 | | -0.37 | | -0.17 | | -0.71 | | -0.77 | |  |  |  |  |
| Probe 14 | | -1.76 | | -0.49 | 0.18 | 0.83 | | 0.90 | | 0.13 | | -0.47 | | 0.68 | | 0.54 | | 1.57 | | -0.58 | | 0.79 | | -0.78 | | 0.26 | | -0.70 | | -1.11 | |  |  |  |  |
| Probe 15 | | -1.45 | | -0.43 | 0.01 | 1.18 | | 0.43 | | 0.26 | | -0.13 | | 0.14 | | 0.55 | | 1.30 | | -0.28 | | 0.53 | | -0.49 | | 0.06 | | -0.72 | | -0.96 | |  |  |  |  |
| Probe 16 | | -1.77 | | -0.46 | -0.36 | 1.32 | | 0.89 | | 0.14 | | -0.40 | | 0.64 | | 0.51 | | 1.25 | | -0.21 | | 0.62 | | -0.63 | | 0.25 | | -0.79 | | -1.01 | |  |  |  |  |
| Probe 17 | | -1.61 | | -0.64 | -0.11 | 1.05 | | 0.58 | | 0.13 | | 0.24 | | 0.36 | | 0.40 | | 1.08 | | -0.42 | | 0.67 | | -0.42 | | 0.20 | | -0.74 | | -0.77 | |  |  |  |  |
| Probe 18 | | -1.62 | | -0.32 | -0.44 | 1.14 | | 0.20 | | 0.33 | | 0.34 | | 0.36 | | 0.15 | | 1.23 | | -0.64 | | 0.85 | | -0.32 | | 0.19 | | -0.85 | | -0.61 | |  |  |  |  |
| Probe 19 | | -1.75 | | -0.52 | 0.01 | 1.05 | | 0.91 | | 0.07 | | 0.02 | | 0.22 | | 0.43 | | 1.01 | | -0.27 | | 0.71 | | -0.85 | | 0.35 | | -0.74 | | -0.63 | |  |  |  |  |
| Probe 20 | | -1.81 | | -0.44 | 0.04 | 1.00 | | 0.48 | | 0.03 | | 0.32 | | 0.39 | | 0.53 | | 1.24 | | -0.55 | | 0.76 | | -0.60 | | 0.27 | | -0.84 | | -0.81 | |  |  |  |  |
| Probe 21 | | -1.58 | | -0.49 | 0.27 | 0.82 | | 0.54 | | -0.08 | | 0.27 | | 0.24 | | 0.48 | | 1.05 | | -0.22 | | 0.63 | | -0.71 | | 0.30 | | -0.84 | | -0.69 | |  |  |  |  |
| Probe 22 | | -1.97 | | -0.74 | 0.21 | 1.11 | | 0.45 | | 0.34 | | 0.24 | | 0.37 | | 0.19 | | 0.95 | | -0.14 | | 0.86 | | -0.51 | | 0.37 | | -0.96 | | -0.76 | |  |  |  |  |
| Probe 23 | | -1.75 | | -0.48 | -0.38 | 0.81 | | 0.68 | | 0.53 | | 0.23 | | 0.36 | | 0.26 | | 1.09 | | -0.28 | | 1.12 | | -0.51 | | 0.15 | | -1.00 | | -0.84 | |  |  |  |  |
| Probe 24 | | -1.72 | | -0.69 | -0.66 | 0.91 | | 0.25 | | 0.51 | | 0.79 | | 0.62 | | 0.31 | | 1.17 | | -0.30 | | 1.06 | | -0.68 | | 0.25 | | -0.97 | | -0.84 | |  |  |  |  |
| Probe 25 | | -1.73 | | -0.56 | -0.47 | 1.09 | | 0.02 | | 0.73 | | 0.27 | | 0.64 | | 0.41 | | 0.95 | | -0.70 | | 0.99 | | -0.40 | | 0.08 | | -0.46 | | -0.86 | |  |  |  |  |
| Probe 26 | | -1.85 | | -0.57 | -0.16 | 0.97 | | 0.47 | | 0.43 | | 0.19 | | 0.52 | | 0.16 | | 1.09 | | -0.50 | | 0.95 | | -0.53 | | 0.18 | | -0.64 | | -0.71 | |  |  |  |  |
| Probe 27 | | -1.72 | | -0.59 | -0.37 | 0.98 | | 0.35 | | 0.37 | | 0.51 | | 0.48 | | 0.10 | | 1.14 | | -0.59 | | 0.97 | | -0.74 | | 0.44 | | -0.88 | | -0.43 | |  |  |  |  |
| Probe 28 | | -1.50 | | -0.52 | -0.42 | 0.95 | | 0.29 | | 0.69 | | 0.09 | | 0.42 | | 0.30 | | 0.85 | | -0.51 | | 0.92 | | -0.48 | | -0.01 | | -0.80 | | -0.28 | |  |  |  |  |
| Probe 29 | | -1.67 | | -0.34 | -0.17 | 1.06 | | 0.13 | | 0.68 | | -0.11 | | 0.43 | | -0.06 | | 0.79 | | -0.39 | | 1.00 | | -0.50 | | 0.20 | | -0.44 | | -0.60 | |  |  |  |  |
| Probe 30 | | -1.64 | | -0.60 | -0.64 | 1.02 | | 0.53 | | 0.74 | | 0.44 | | 0.16 | | -0.09 | | 0.92 | | -0.38 | | 1.10 | | -0.69 | | 0.18 | | -0.77 | | -0.27 | |  |  |  |  |
| Probe 31 | | -1.49 | | -0.56 | -0.26 | 1.13 | | 0.48 | | 0.28 | | 0.19 | | 0.23 | | 0.20 | | 0.75 | | -0.34 | | 0.86 | | -0.63 | | 0.28 | | -0.81 | | -0.31 | |  |  |  |  |
| Probe 32 | | -1.58 | | -0.73 | -0.13 | 0.94 | | 0.32 | | 0.55 | | 0.05 | | 0.59 | | 0.10 | | 0.74 | | -0.41 | | 1.03 | | -0.53 | | 0.38 | | -0.64 | | -0.68 | |  |  |  |  |
| Probe 33 | | -1.39 | | -0.64 | -0.30 | 1.01 | | 0.32 | | 0.45 | | 0.43 | | 0.12 | | 0.08 | | 0.64 | | -0.43 | | 0.94 | | -0.28 | | 0.42 | | -0.81 | | -0.56 | |  |  |  |  |
| Probe 34 | | -1.44 | | -0.42 | -0.31 | 0.89 | | 0.43 | | 0.52 | | 0.37 | | -0.04 | | 0.12 | | 0.76 | | -0.40 | | 0.95 | | -0.38 | | 0.13 | | -0.87 | | -0.31 | |  |  |  |  |
| Probe 35 | | -1.60 | | -0.39 | 0.20 | 0.81 | | 0.23 | | 0.27 | | 0.34 | | 0.15 | | -0.22 | | 0.73 | | -0.61 | | 1.06 | | -0.65 | | 0.43 | | -0.67 | | -0.06 | |  |  |  |  |
| Probe 36 | | -1.24 | | -0.57 | -0.48 | 0.85 | | 0.34 | | 0.43 | | 0.54 | | 0.14 | | 0.16 | | 0.66 | | -0.37 | | 1.03 | | -0.64 | | 0.17 | | -0.73 | | -0.29 | |  |  |  |  |
| Probe 37 | | -1.47 | | -0.63 | 0.11 | 0.68 | | 0.63 | | 0.39 | | 0.13 | | 0.17 | | -0.04 | | 0.40 | | -0.55 | | 0.98 | | -0.63 | | 0.37 | | -0.52 | | -0.02 | |  |  |  |  |
| Probe 38 | | -1.50 | | -0.54 | -0.71 | 1.04 | | 0.36 | | 0.63 | | 0.46 | | 0.26 | | -0.13 | | 0.58 | | -1.05 | | 1.12 | | -0.15 | | 0.40 | | -0.62 | | -0.14 | |  |  |  |  |
| Probe 39 | | -1.22 | | -0.47 | -0.41 | 1.03 | | 0.07 | | 0.33 | | 0.44 | | 0.23 | | 0.09 | | 0.72 | | -0.60 | | 0.88 | | -0.24 | | 0.35 | | -0.88 | | -0.32 | |  |  |  |  |
| Probe 40 | | -1.57 | | -0.54 | -0.18 | 0.86 | | 0.31 | | 0.33 | | 0.38 | | 0.41 | | -0.21 | | 0.44 | | -0.52 | | 1.05 | | -0.19 | | 0.18 | | -0.59 | | -0.15 | |  |  |  |  |
| Probe 41 | | -0.99 | | -0.53 | -0.10 | 0.64 | | 0.21 | | 0.32 | | 0.17 | | 0.28 | | 0.16 | | 0.50 | | -0.53 | | 1.06 | | -0.23 | | 0.34 | | -1.12 | | -0.19 | |  |  |  |  |
| Probe 42 | | -0.99 | | -0.36 | -0.34 | 0.60 | | 0.02 | | 0.30 | | 0.57 | | 0.19 | | 0.11 | | 0.53 | | -0.90 | | 1.24 | | -0.25 | | 0.47 | | -1.03 | | -0.17 | |  |  |  |  |
| Probe 43 | | -1.07 | | -0.45 | -0.82 | 0.87 | | 0.44 | | 0.37 | | 0.27 | | 0.39 | | 0.05 | | 0.49 | | -1.18 | | 1.18 | | -0.39 | | 0.48 | | -0.72 | | 0.10 | |  |  |  |  |
| Probe 44 | | -1.34 | | -0.25 | -0.25 | 0.76 | | 0.29 | | 0.24 | | 0.41 | | 0.14 | | -0.11 | | 0.70 | | -0.87 | | 1.14 | | -0.53 | | 0.41 | | -0.85 | | 0.11 | |  |  |  |  |
| Probe 45 | | -1.11 | | -0.15 | -0.31 | 0.84 | | 0.49 | | 0.13 | | -0.14 | | 0.24 | | 0.18 | | 0.57 | | -0.93 | | 1.07 | | -0.50 | | 0.27 | | -0.52 | | -0.15 | |  |  |  |  |
| Supplementary Table 6: 2P NRM parameter estimates for multinomial ratings in the Arrow Flanker for Kane et al. (2016) | | | | | | | | | | | | | | | | | | | | | | | | | | | | | | | | | | | |
| Probe Num. | | *a_1_* | | *a_2_* | | *a_3_* | | *a_4_* | | *a_5_* | | *a_6_* | | *a_7_* | | *a_8_* | | *c_1_* | | *c_2_* | | *c_3_* | | *c_4_* | | *c_5_* | | *c_6_* | | *c_7_* | | *c_8_* | | |  |
| Probe 1 | | -1.02 | | -0.48 | | 0.09 | | 0.36 | | 0.11 | | 0.73 | | 0.05 | | 0.15 | | 2.35 | | 1.41 | | -0.25 | | 0.62 | | -0.77 | | -0.45 | | -1.43 | | -1.48 | | |  |
| Probe 2 | | -1.29 | | -0.67 | | -0.42 | | 0.33 | | 0.51 | | 1.09 | | 0.18 | | 0.27 | | 1.55 | | 1.13 | | -0.09 | | 0.92 | | -0.37 | | -0.58 | | -1.57 | | -0.99 | | |  |
| Probe 3 | | -1.93 | | -0.64 | | -0.27 | | 0.62 | | 0.67 | | 1.27 | | -0.36 | | 0.63 | | 0.94 | | 1.00 | | 0.21 | | 0.98 | | -0.13 | | -0.53 | | -1.68 | | -0.80 | | |  |
| Probe 4 | | -1.90 | | -0.48 | | -0.23 | | 0.25 | | 0.49 | | 0.91 | | 0.19 | | 0.77 | | 0.77 | | 0.96 | | 0.07 | | 1.09 | | 0.10 | | -0.05 | | -1.79 | | -1.14 | | |  |
| Probe 5 | | -2.26 | | -0.60 | | 0.02 | | 0.29 | | 0.56 | | 0.98 | | 0.24 | | 0.76 | | 0.24 | | 0.73 | | -0.02 | | 1.16 | | 0.04 | | 0.13 | | -1.15 | | -1.13 | | |  |
| Probe 6 | | -2.04 | | -1.07 | | -0.25 | | 0.32 | | 0.38 | | 1.31 | | 0.90 | | 0.45 | | 1.09 | | 0.89 | | 0.18 | | 1.03 | | 0.20 | | -0.34 | | -1.91 | | -1.13 | | |  |
| Probe 7 | | -1.87 | | -1.04 | | -0.25 | | 0.50 | | 0.43 | | 1.24 | | 1.01 | | -0.02 | | 1.26 | | 0.94 | | 0.08 | | 0.91 | | -0.26 | | -0.47 | | -1.80 | | -0.65 | | |  |
| Probe 8 | | -2.19 | | -0.91 | | -0.52 | | 0.32 | | 0.65 | | 1.32 | | 1.36 | | -0.04 | | 1.16 | | 1.09 | | 0.28 | | 1.21 | | -0.28 | | -0.48 | | -2.31 | | -0.68 | | |  |
| Probe 9 | | -2.00 | | -0.85 | | -0.31 | | 0.45 | | 0.22 | | 1.04 | | 1.08 | | 0.37 | | 1.16 | | 0.84 | | -0.11 | | 0.96 | | 0.16 | | -0.10 | | -1.88 | | -1.03 | | |  |
| Probe 10 | | -2.06 | | -0.95 | | -0.48 | | 0.46 | | 0.46 | | 0.93 | | 0.98 | | 0.67 | | 1.12 | | 0.75 | | 0.04 | | 0.83 | | -0.08 | | -0.06 | | -1.74 | | -0.86 | | |  |
| Probe 11 | | -2.44 | | -1.05 | | -0.54 | | 0.21 | | 0.76 | | 1.33 | | 0.94 | | 0.79 | | 1.15 | | 1.12 | | -0.20 | | 1.14 | | -0.15 | | -0.35 | | -1.46 | | -1.26 | | |  |
| Probe 12 | | -2.29 | | -0.91 | | -0.59 | | 0.44 | | 0.85 | | 1.31 | | 0.54 | | 0.65 | | 1.22 | | 0.97 | | 0.28 | | 0.99 | | -0.46 | | -0.29 | | -1.42 | | -1.28 | | |  |
| Probe 13 | | -2.57 | | -1.00 | | -0.40 | | 0.60 | | 0.89 | | 1.80 | | -0.04 | | 0.71 | | 0.81 | | 1.02 | | 0.11 | | 0.99 | | -0.21 | | -0.65 | | -0.89 | | -1.18 | | |  |
| Probe 14 | | -2.33 | | -1.21 | | -0.57 | | 0.39 | | 0.65 | | 1.66 | | 0.65 | | 0.77 | | 1.19 | | 0.90 | | 0.19 | | 1.08 | | -0.29 | | -0.65 | | -1.46 | | -0.95 | | |  |
| Probe 15 | | -2.33 | | -0.95 | | -0.62 | | 0.51 | | 0.27 | | 1.59 | | 0.49 | | 1.04 | | 1.23 | | 1.05 | | -0.02 | | 1.13 | | -0.22 | | -0.35 | | -1.57 | | -1.25 | | |  |
| Probe 16 | | -2.29 | | -1.04 | | -0.08 | | 0.53 | | 0.81 | | 1.52 | | -0.25 | | 0.80 | | 0.95 | | 0.92 | | 0.00 | | 0.90 | | -0.53 | | -0.38 | | -1.08 | | -0.79 | | |  |
| Probe 17 | | -1.85 | | -0.81 | | -0.22 | | 0.34 | | 0.61 | | 1.24 | | 0.55 | | 0.13 | | 1.00 | | 0.92 | | -0.14 | | 0.84 | | -0.61 | | -0.51 | | -0.96 | | -0.55 | | |  |
| Probe 18 | | -2.22 | | -0.91 | | -0.29 | | 0.38 | | 0.64 | | 1.27 | | 0.68 | | 0.47 | | 1.06 | | 0.80 | | 0.08 | | 1.04 | | -0.99 | | -0.49 | | -0.90 | | -0.59 | | |  |
| Probe 19 | | -2.51 | | -1.35 | | -0.54 | | 0.52 | | 0.77 | | 1.70 | | 0.45 | | 0.96 | | 1.25 | | 0.71 | | 0.00 | | 1.02 | | -0.46 | | -0.65 | | -0.69 | | -1.19 | | |  |
| Probe 20 | | -2.06 | | -1.06 | | -0.05 | | 0.63 | | 0.73 | | 1.77 | | -0.71 | | 0.76 | | 1.13 | | 0.72 | | -0.15 | | 1.00 | | -0.38 | | -0.66 | | -0.64 | | -1.01 | | |  |

| Supplementary Table 7: 2P NRM parameter estimates for multinomial ratings in the Number Stroop for Kane et al. (2016) | | | | | | | | | | | | | | | | | |
| --- | --- | --- | --- | --- | --- | --- | --- | --- | --- | --- | --- | --- | --- | --- | --- | --- | --- |
| Probe Num. | *a_1_* | *a_2_* | *a_3_* | *a_4_* | *a_5_* | *a_6_* | *a_7_* | *a_8_* | *c_1_* | *c_2_* | *c_3_* | *c_4_* | *c_5_* | *c_6_* | *c_7_* | *c_8_* |  |
| Probe 1 | -0.88 | -0.57 | -0.26 | 0.79 | 0.51 | 0.60 | -0.21 | 0.03 | 1.01 | 1.01 | 0.36 | 0.89 | -0.26 | -0.12 | -1.39 | -1.50 |  |
| Probe 2 | -1.26 | -0.56 | 0.02 | 1.22 | 0.39 | 1.00 | -0.52 | -0.29 | 1.42 | 1.10 | 0.01 | 0.63 | -0.35 | -0.39 | -1.17 | -1.25 |  |
| Probe 3 | -1.21 | -0.79 | -0.01 | 0.87 | 0.15 | 0.66 | 0.40 | -0.06 | 1.39 | 1.15 | -0.02 | 0.76 | -0.19 | -0.52 | -1.66 | -0.92 |  |
| Probe 4 | -1.61 | -0.88 | 0.15 | 1.14 | 0.29 | 0.91 | 0.08 | -0.08 | 1.33 | 1.07 | 0.20 | 0.67 | -0.53 | -0.65 | -1.28 | -0.81 |  |
| Probe 5 | -1.79 | -0.94 | 0.23 | 1.32 | 0.06 | 0.72 | 0.12 | 0.28 | 1.23 | 1.10 | -0.27 | 0.44 | -0.15 | 0.07 | -1.19 | -1.23 |  |
| Probe 6 | -1.53 | -0.83 | -0.30 | 1.16 | 0.43 | 0.70 | -0.02 | 0.39 | 1.38 | 1.22 | -0.13 | 0.50 | -0.22 | -0.31 | -1.06 | -1.37 |  |
| Probe 7 | -1.67 | -1.02 | -0.08 | 1.60 | 0.94 | 1.15 | -1.14 | 0.22 | 1.44 | 1.34 | -0.21 | 0.60 | -0.30 | -0.38 | -1.47 | -1.04 |  |
| Probe 8 | -1.87 | -0.87 | -0.42 | 1.43 | 0.24 | 1.21 | 0.22 | 0.07 | 1.11 | 1.20 | -0.10 | 0.49 | 0.03 | -0.49 | -1.41 | -0.83 |  |
| Probe 9 | -1.85 | -0.91 | -0.16 | 1.38 | 0.57 | 1.17 | -0.68 | 0.47 | 1.34 | 1.30 | -0.25 | 0.57 | -0.11 | -0.23 | -1.55 | -1.08 |  |
| Probe 10 | -1.65 | -0.83 | 0.21 | 1.33 | 0.50 | 1.13 | -0.54 | -0.15 | 1.12 | 1.11 | -0.06 | 0.59 | -0.77 | -0.37 | -0.79 | -0.84 |  |
| Probe 11 | -1.95 | -1.09 | -0.13 | 1.59 | 1.03 | 1.06 | -0.64 | 0.13 | 1.09 | 1.04 | -0.12 | 0.49 | -0.62 | -0.26 | -0.97 | -0.65 |  |
| Probe 12 | -1.55 | -1.03 | -0.06 | 1.48 | 0.93 | 1.00 | -0.36 | -0.41 | 1.34 | 1.12 | -0.07 | 0.62 | -0.87 | -0.21 | -1.09 | -0.83 |  |
| Probe 13 | -1.77 | -0.91 | -0.07 | 1.72 | 0.62 | 0.98 | -0.64 | 0.06 | 1.15 | 1.32 | -0.43 | 0.34 | -0.32 | -0.30 | -1.03 | -0.73 |  |
| Probe 14 | -2.15 | -1.02 | -0.22 | 1.65 | 1.37 | 1.09 | -0.30 | -0.41 | 1.01 | 1.19 | 0.16 | 0.32 | -0.91 | -0.13 | -0.78 | -0.85 |  |
| Probe 15 | -1.69 | -0.93 | -0.11 | 1.60 | 0.76 | 0.73 | -0.12 | -0.24 | 1.25 | 1.09 | -0.10 | 0.10 | -0.54 | -0.16 | -1.10 | -0.54 |  |
| Probe 16 | -2.30 | -0.91 | 0.22 | 1.79 | 1.26 | 1.12 | -0.85 | -0.33 | 0.91 | 1.22 | 0.11 | 0.51 | -0.53 | -0.15 | -1.34 | -0.73 |  |
| Probe 17 | -1.86 | -1.08 | -0.03 | 1.55 | 0.79 | 1.06 | 0.04 | -0.46 | 1.14 | 1.07 | 0.02 | 0.18 | -0.54 | -0.25 | -1.03 | -0.59 |  |
| Probe 18 | -1.77 | -0.98 | -0.29 | 1.52 | 0.71 | 0.69 | 0.33 | -0.22 | 1.12 | 1.00 | -0.19 | 0.38 | -0.24 | -0.06 | -1.09 | -0.93 |  |
| Probe 19 | -1.42 | -0.64 | -0.28 | 1.28 | 0.62 | 0.76 | -0.21 | -0.11 | 1.16 | 0.97 | -0.22 | 0.56 | -0.53 | -0.19 | -0.91 | -0.84 |  |
| Probe 20 | -1.17 | -0.55 | -0.02 | 1.19 | 0.39 | 0.87 | -0.43 | -0.27 | 1.33 | 1.04 | -0.28 | 0.54 | -0.62 | -0.34 | -0.71 | -0.97 |  |

| Supplementary Table 8: 2P NRM parameter estimates for multinomial ratings in the Letter Flanker for Kane et al. (2016) | | | | | | | | | | | | | | | | | |
| --- | --- | --- | --- | --- | --- | --- | --- | --- | --- | --- | --- | --- | --- | --- | --- | --- | --- |
| Probe Num. | *a_1_* | *a_2_* | *a_3_* | *a_4_* | *a_5_* | *a_6_* | *a_7_* | *a_8_* | *c_1_* | *c_2_* | *c_3_* | *c_4_* | *c_5_* | *c_6_* | *c_7_* | *c_8_* |  |
| Probe 1 | -0.61 | -0.11 | -0.47 | 1.04 | -0.42 | 0.17 | -0.35 | 0.76 | 2.35 | 1.18 | -0.17 | 0.45 | -0.86 | -0.31 | -1.36 | -1.30 |  |
| Probe 2 | -0.87 | -0.15 | 0.01 | 0.67 | 0.09 | -0.02 | -0.33 | 0.60 | 1.40 | 0.87 | -0.24 | 0.82 | -0.59 | -0.39 | -0.57 | -1.30 |  |
| Probe 3 | -1.14 | -0.27 | -0.21 | 1.23 | 0.06 | 0.64 | -0.70 | 0.39 | 1.06 | 0.74 | 0.20 | 0.59 | -0.19 | -0.21 | -1.04 | -1.16 |  |
| Probe 4 | -1.06 | 0.02 | 0.01 | 0.92 | 0.28 | 0.07 | -0.16 | -0.08 | 0.76 | 0.46 | -0.20 | 0.65 | -0.32 | -0.02 | -0.67 | -0.66 |  |
| Probe 5 | -1.20 | -0.23 | -0.14 | 1.37 | 0.12 | 0.36 | -0.46 | 0.18 | 0.78 | 0.44 | -0.15 | 0.65 | -0.42 | -0.07 | -0.48 | -0.74 |  |
| Probe 6 | -1.20 | -0.61 | 0.01 | 1.04 | -0.18 | 0.32 | -0.16 | 0.77 | 0.64 | 0.18 | -0.21 | 0.91 | -0.16 | 0.29 | -0.79 | -0.86 |  |
| Probe 7 | -1.27 | -0.37 | 0.00 | 1.18 | 0.03 | 0.37 | -0.29 | 0.35 | 0.66 | 0.16 | -0.15 | 0.65 | -0.04 | 0.30 | -0.54 | -1.04 |  |
| Probe 8 | -1.50 | -0.43 | 0.15 | 0.96 | 0.34 | 0.41 | -0.07 | 0.15 | 0.39 | 0.28 | -0.06 | 0.88 | -0.46 | 0.10 | -0.45 | -0.68 |  |
| Probe 9 | -1.33 | -0.81 | -0.18 | 0.96 | 0.13 | 0.48 | 0.01 | 0.74 | 0.43 | 0.07 | -0.05 | 0.98 | -0.03 | 0.18 | -0.50 | -1.08 |  |
| Probe 10 | -1.51 | -0.44 | -0.14 | 1.23 | 0.38 | 0.28 | -0.19 | 0.38 | 0.06 | 0.29 | 0.06 | 0.96 | -0.34 | 0.22 | -0.77 | -0.49 |  |
| Probe 11 | -1.16 | -0.45 | 0.05 | 1.12 | 0.18 | 0.17 | -0.51 | 0.61 | 0.49 | 0.01 | -0.05 | 0.86 | -0.23 | 0.32 | -0.63 | -0.76 |  |
| Probe 12 | -0.83 | -0.38 | 0.12 | 0.66 | -0.16 | 0.31 | 0.06 | 0.23 | 0.53 | 0.04 | -0.28 | 1.07 | -0.20 | 0.23 | -0.55 | -0.84 |  |

| Supplementary Table 9: 2P NRM parameter estimates for multinomial ratings in the *N*-Back for Kane et al. (2016) | | | | | | | | | | | | | | | | | |
| --- | --- | --- | --- | --- | --- | --- | --- | --- | --- | --- | --- | --- | --- | --- | --- | --- | --- |
| Probe Num. | *a_1_* | *a_2_* | *a_3_* | *a_4_* | *a_5_* | *a_6_* | *a_7_* | *a_8_* | *c_1_* | *c_2_* | *c_3_* | *c_4_* | *c_5_* | *c_6_* | *c_7_* | *c_8_* |  |
| Probe 1 | -1.18 | -0.77 | 0.31 | 0.39 | 1.02 | 1.52 | -0.72 | -0.56 | 3.13 | 1.80 | -0.76 | 0.45 | -0.94 | -1.89 | -0.60 | -1.18 |  |
| Probe 2 | -1.97 | -1.08 | -0.28 | 0.38 | 1.45 | 1.94 | 0.09 | -0.53 | 2.64 | 2.33 | 0.26 | 0.78 | -1.47 | -2.01 | -0.98 | -1.55 |  |
| Probe 3 | -2.15 | -1.36 | -0.01 | 0.09 | 0.89 | 1.55 | 0.75 | 0.23 | 2.25 | 2.25 | -0.04 | 1.00 | -0.68 | -1.41 | -1.80 | -1.57 |  |
| Probe 4 | -2.63 | -1.34 | -0.67 | 0.32 | 0.82 | 1.80 | 0.97 | 0.72 | 1.79 | 1.77 | 0.38 | 1.10 | -0.24 | -1.03 | -1.64 | -2.14 |  |
| Probe 5 | -2.91 | -1.78 | -0.32 | 0.44 | 1.35 | 2.03 | 1.14 | 0.05 | 2.24 | 2.13 | -0.10 | 0.87 | -0.79 | -1.29 | -1.61 | -1.43 |  |
| Probe 6 | -2.74 | -1.39 | 0.05 | 0.25 | 0.87 | 1.38 | 0.33 | 1.25 | 1.18 | 1.64 | -0.02 | 1.37 | -0.31 | -0.33 | -1.52 | -2.02 |  |
| Probe 7 | -2.28 | -1.29 | 0.01 | 0.52 | 0.74 | 1.46 | -0.09 | 0.94 | 1.22 | 1.36 | -0.05 | 1.05 | -0.24 | -0.44 | -1.33 | -1.58 |  |
| Probe 8 | -2.59 | -1.67 | -0.70 | 0.36 | 0.41 | 2.22 | 0.43 | 1.55 | 2.22 | 1.87 | 0.57 | 1.26 | -0.08 | -1.72 | -2.13 | -1.99 |  |
| Probe 9 | -2.58 | -1.30 | 0.02 | 0.00 | 0.66 | 1.35 | 0.96 | 0.90 | 1.53 | 1.39 | 0.05 | 1.23 | -0.35 | -0.54 | -1.68 | -1.63 |  |
| Probe 10 | -2.73 | -1.41 | -0.29 | 0.34 | 1.20 | 1.18 | 0.28 | 1.43 | 1.22 | 1.24 | -0.22 | 1.37 | -0.57 | -0.11 | -1.26 | -1.68 |  |
| Probe 11 | -1.82 | -0.73 | -0.05 | 0.30 | 0.57 | 1.18 | 0.09 | 0.46 | 0.80 | 0.84 | -0.69 | 1.33 | -0.15 | -0.06 | -1.18 | -0.89 |  |
| Probe 12 | -1.96 | -0.67 | -0.19 | 0.41 | 0.60 | 1.29 | 0.05 | 0.47 | 0.52 | 0.86 | -0.40 | 1.12 | -0.33 | -0.20 | -0.67 | -0.91 |  |
| Probe 13 | -1.88 | -0.77 | -0.63 | 0.13 | 1.12 | 1.00 | 0.50 | 0.53 | 1.54 | 1.10 | -0.05 | 1.31 | -1.31 | -0.25 | -1.38 | -0.96 |  |
| Probe 14 | -1.99 | -1.04 | 0.19 | -0.03 | 0.83 | 1.35 | 0.33 | 0.37 | 1.14 | 1.16 | -0.99 | 1.51 | -0.47 | -0.14 | -1.14 | -1.06 |  |
| Probe 15 | -1.93 | -0.84 | -0.18 | 0.08 | 0.64 | 0.95 | 0.97 | 0.31 | 0.83 | 0.75 | -0.34 | 1.34 | -0.48 | -0.12 | -1.37 | -0.61 |  |

| Supplementary Table 10: 2PL parameter estimates for dichotomized ratings in the SART for Kane et al. (2016) | | | | |
| --- | --- | --- | --- | --- |
| Probe Num. | *a* | *b* | Information | % Total Information |
| Probe 1 | 0.87 | 2.74 | 0.87 | 1.31 |
| Probe 2 | 1.07 | 2.00 | 1.07 | 1.61 |
| Probe 3 | 1.05 | 1.64 | 1.05 | 1.58 |
| Probe 4 | 1.17 | 1.03 | 1.17 | 1.75 |
| Probe 5 | 1.11 | 1.19 | 1.11 | 1.67 |
| Probe 6 | 1.11 | 0.86 | 1.11 | 1.67 |
| Probe 7 | 1.09 | 0.87 | 1.09 | 1.64 |
| Probe 8 | 1.27 | 0.51 | 1.27 | 1.91 |
| Probe 9 | 1.44 | 0.48 | 1.44 | 2.17 |
| Probe 10 | 1.74 | 0.48 | 1.74 | 2.61 |
| Probe 11 | 1.41 | 0.52 | 1.41 | 2.12 |
| Probe 12 | 1.61 | 0.30 | 1.61 | 2.43 |
| Probe 13 | 1.75 | 0.26 | 1.75 | 2.63 |
| Probe 14 | 1.73 | 0.16 | 1.73 | 2.61 |
| Probe 15 | 1.55 | 0.07 | 1.55 | 2.33 |
| Probe 16 | 1.73 | -0.01 | 1.73 | 2.61 |
| Probe 17 | 1.85 | -0.11 | 1.85 | 2.79 |
| Probe 18 | 1.58 | -0.14 | 1.58 | 2.37 |
| Probe 19 | 1.78 | -0.14 | 1.78 | 2.68 |
| Probe 20 | 1.69 | 0.01 | 1.69 | 2.54 |
| Probe 21 | 1.66 | -0.10 | 1.66 | 2.49 |
| Probe 22 | 2.21 | -0.22 | 2.21 | 3.32 |
| Probe 23 | 1.86 | -0.21 | 1.86 | 2.79 |
| Probe 24 | 1.79 | -0.18 | 1.79 | 2.70 |
| Probe 25 | 1.69 | -0.21 | 1.69 | 2.54 |
| Probe 26 | 1.71 | -0.19 | 1.71 | 2.58 |
| Probe 27 | 1.65 | -0.23 | 1.65 | 2.47 |
| Probe 28 | 1.57 | -0.30 | 1.57 | 2.36 |
| Probe 29 | 1.63 | -0.44 | 1.63 | 2.45 |
| Probe 30 | 1.44 | -0.45 | 1.44 | 2.17 |
| Probe 31 | 1.49 | -0.40 | 1.49 | 2.24 |
| Probe 32 | 1.66 | -0.42 | 1.65 | 2.49 |
| Probe 33 | 1.59 | -0.51 | 1.59 | 2.40 |
| Probe 34 | 1.32 | -0.47 | 1.32 | 1.98 |
| Probe 35 | 1.43 | -0.60 | 1.43 | 2.15 |
| Probe 36 | 1.34 | -0.55 | 1.34 | 2.02 |
| Probe 37 | 1.36 | -0.71 | 1.35 | 2.04 |
| Probe 38 | 1.53 | -0.65 | 1.53 | 2.30 |
| Probe 39 | 1.38 | -0.54 | 1.37 | 2.07 |
| Probe 40 | 1.70 | -0.66 | 1.70 | 2.55 |
| Probe 41 | 1.28 | -0.72 | 1.28 | 1.92 |
| Probe 42 | 1.10 | -0.90 | 1.10 | 1.65 |
| Probe 43 | 1.26 | -0.83 | 1.26 | 1.90 |
| Probe 44 | 1.21 | -0.74 | 1.21 | 1.82 |
| Probe 45 | 1.06 | -0.76 | 1.06 | 1.60 |

| Supplementary Table 11: 2PL parameter estimates for dichotomized ratings in the Arrow Flanker for Kane et al. (2016) | | | | |
| --- | --- | --- | --- | --- |
| Probe Num. | *a* | *b* | Information | % Total Information |
| Probe 1 | 1.25 | 1.06 | 1.25 | 3.15 |
| Probe 2 | 1.33 | 0.31 | 1.33 | 3.35 |
| Probe 3 | 1.70 | -0.05 | 1.70 | 4.27 |
| Probe 4 | 1.75 | -0.16 | 1.75 | 4.39 |
| Probe 5 | 2.00 | -0.36 | 2.00 | 5.04 |
| Probe 6 | 2.02 | -0.08 | 2.02 | 5.07 |
| Probe 7 | 1.82 | 0.05 | 1.81 | 4.57 |
| Probe 8 | 1.91 | -0.03 | 1.91 | 4.82 |
| Probe 9 | 1.85 | -0.03 | 1.84 | 4.64 |
| Probe 10 | 2.06 | -0.05 | 2.06 | 5.17 |
| Probe 11 | 1.99 | 0.04 | 1.99 | 5.00 |
| Probe 12 | 2.11 | 0.02 | 2.11 | 5.32 |
| Probe 13 | 2.34 | -0.06 | 2.34 | 5.88 |
| Probe 14 | 2.41 | -0.03 | 2.41 | 6.07 |
| Probe 15 | 1.98 | 0.03 | 1.98 | 4.97 |
| Probe 16 | 2.33 | -0.03 | 2.33 | 5.86 |
| Probe 17 | 1.96 | 0.01 | 1.96 | 4.93 |
| Probe 18 | 2.21 | -0.05 | 2.21 | 5.55 |
| Probe 19 | 2.53 | -0.02 | 2.53 | 6.36 |
| Probe 20 | 2.23 | -0.05 | 2.23 | 5.61 |

| Supplementary Table 12: 2PL parameter estimates for dichotomized ratings in the Number Stroop for Kane et al. (2016) | | | | |
| --- | --- | --- | --- | --- |
| Probe Num. | *a* | *b* | Information | % Total Information |
| Probe 1 | 1.18 | -0.11 | 1.18 | 2.99 |
| Probe 2 | 1.80 | 0.21 | 1.80 | 4.54 |
| Probe 3 | 1.62 | 0.21 | 1.62 | 4.10 |
| Probe 4 | 1.90 | 0.15 | 1.90 | 4.79 |
| Probe 5 | 1.95 | 0.14 | 1.95 | 4.94 |
| Probe 6 | 2.02 | 0.23 | 2.02 | 5.11 |
| Probe 7 | 2.32 | 0.22 | 2.32 | 5.86 |
| Probe 8 | 2.14 | 0.11 | 2.14 | 5.42 |
| Probe 9 | 2.21 | 0.20 | 2.21 | 5.59 |
| Probe 10 | 1.96 | 0.11 | 1.96 | 4.96 |
| Probe 11 | 2.33 | 0.07 | 2.33 | 5.89 |
| Probe 12 | 2.04 | 0.15 | 2.04 | 5.17 |
| Probe 13 | 2.19 | 0.20 | 2.19 | 5.55 |
| Probe 14 | 2.22 | 0.09 | 2.22 | 5.61 |
| Probe 15 | 2.15 | 0.17 | 2.15 | 5.43 |
| Probe 16 | 2.38 | 0.06 | 2.38 | 6.01 |
| Probe 17 | 2.03 | 0.13 | 2.03 | 5.14 |
| Probe 18 | 2.02 | 0.08 | 2.02 | 5.12 |
| Probe 19 | 1.62 | 0.10 | 1.62 | 4.10 |
| Probe 20 | 1.46 | 0.22 | 1.46 | 3.69 |

| Supplementary Table 13: 2PL parameter estimates for dichotomized ratings in the Letter Flanker for Kane et al. (2016) | | | | |
| --- | --- | --- | --- | --- |
| Probe Num. | *a* | *b* | Information | % Total Information |
| Probe 1 | 1.15 | 1.03 | 1.15 | 7.12 |
| Probe 2 | 1.20 | 0.21 | 1.20 | 7.43 |
| Probe 3 | 1.35 | (0.09) | 1.35 | 8.33 |
| Probe 4 | 1.25 | (0.31) | 1.25 | 7.71 |
| Probe 5 | 1.67 | (0.29) | 1.67 | 10.29 |
| Probe 6 | 1.56 | (0.51) | 1.56 | 9.66 |
| Probe 7 | 1.29 | (0.52) | 1.29 | 7.95 |
| Probe 8 | 1.32 | (0.56) | 1.32 | 8.15 |
| Probe 9 | 1.54 | (0.63) | 1.54 | 9.50 |
| Probe 10 | 1.44 | (0.71) | 1.44 | 8.89 |
| Probe 11 | 1.41 | (0.69) | 1.41 | 8.71 |
| Probe 12 | 1.01 | (0.91) | 1.01 | 6.25 |

| Supplementary Table 14: 2PL parameter estimates for dichotomized ratings in the *N*-Back for Kane et al. (2016) | | | | |
| --- | --- | --- | --- | --- |
| Probe Num. | *a* | *b* | Information | % Total Information |
| Probe 1 | 1.57 | 1.40 | 1.57 | 4.73 |
| Probe 2 | 2.05 | 0.90 | 2.05 | 6.18 |
| Probe 3 | 2.26 | 0.67 | 2.26 | 6.81 |
| Probe 4 | 2.19 | 0.32 | 2.19 | 6.61 |
| Probe 5 | 2.88 | 0.50 | 2.88 | 8.69 |
| Probe 6 | 2.58 | 0.11 | 2.58 | 7.79 |
| Probe 7 | 2.44 | 0.11 | 2.44 | 7.35 |
| Probe 8 | 2.73 | 0.35 | 2.73 | 8.22 |
| Probe 9 | 2.52 | 0.16 | 2.52 | 7.60 |
| Probe 10 | 2.62 | 0.02 | 2.62 | 7.90 |
| Probe 11 | 1.89 | -0.21 | 1.89 | 5.71 |
| Probe 12 | 1.97 | -0.21 | 1.97 | 5.94 |
| Probe 13 | 1.86 | 0.12 | 1.86 | 5.62 |
| Probe 14 | 1.83 | -0.06 | 1.83 | 5.53 |
| Probe 15 | 1.76 | -0.24 | 1.76 | 5.31 |

| Supplementary Table 15: 2PL parameter estimates for Zanesco et al. (2020) | | | | |
| --- | --- | --- | --- | --- |
| Probe Num. | a | b | Information | % Total Information |
| Probe 1 | 2.35 | 1.17 | 2.35 | 2.47 |
| Probe 2 | 1.73 | 0.88 | 1.73 | 1.81 |
| Probe 3 | 2.24 | 0.61 | 2.24 | 2.35 |
| Probe 4 | 2.93 | 1.11 | 2.93 | 3.07 |
| Probe 5 | 2.07 | 0.27 | 2.07 | 2.16 |
| Probe 6 | 2.03 | 0.32 | 2.03 | 2.13 |
| Probe 7 | 2.34 | 0.54 | 2.34 | 2.45 |
| Probe 8 | 2.41 | 0.55 | 2.41 | 2.52 |
| Probe 9 | 2.41 | 0.25 | 2.41 | 2.53 |
| Probe 10 | 3.37 | 0.81 | 3.37 | 3.53 |
| Probe 11 | 2.82 | 0.13 | 2.82 | 2.95 |
| Probe 12 | 4.42 | 0.35 | 4.42 | 4.63 |
| Probe 13 | 4.47 | 0.63 | 4.47 | 4.69 |
| Probe 14 | 4.64 | 0.67 | 4.64 | 4.86 |
| Probe 15 | 4.63 | 0.70 | 4.63 | 4.85 |
| Probe 16 | 4.65 | 0.69 | 4.65 | 4.88 |
| Probe 17 | 3.17 | 0.30 | 3.17 | 3.32 |
| Probe 18 | 3.22 | 0.20 | 3.22 | 3.37 |
| Probe 19 | 4.32 | 0.69 | 4.32 | 4.52 |
| Probe 20 | 3.56 | 0.32 | 3.56 | 3.73 |
| Probe 21 | 3.14 | 0.11 | 3.14 | 3.29 |
| Probe 22 | 3.86 | 0.27 | 3.86 | 4.05 |
| Probe 23 | 4.87 | 0.45 | 4.87 | 5.10 |
| Probe 24 | 4.18 | 0.24 | 4.18 | 4.38 |
| Probe 25 | 5.00 | 0.43 | 5.00 | 5.24 |
| Probe 26 | 4.17 | 0.20 | 4.17 | 4.37 |
| Probe 27 | 3.74 | 0.17 | 3.74 | 3.92 |
| Probe 28 | 2.69 | -0.02 | 2.69 | 2.81 |

| Supplementary Table 16: 2PL parameter estimates for Goller et al. (2020) | | | | |
| --- | --- | --- | --- | --- |
| Probe Num. | a | b | Information | % Total Information |
| Probe 1 | 1.08 | 0.12 | 1.08 | 1.29 |
| Probe 2 | 1.19 | -0.32 | 1.19 | 1.42 |
| Probe 3 | 1.43 | -0.25 | 1.43 | 1.71 |
| Probe 4 | 1.35 | -0.13 | 1.35 | 1.62 |
| Probe 5 | 1.54 | -0.54 | 1.54 | 1.84 |
| Probe 6 | 1.76 | -0.31 | 1.76 | 2.11 |
| Probe 7 | 2.45 | -0.23 | 2.45 | 2.93 |
| Probe 8 | 2.12 | -0.47 | 2.12 | 2.54 |
| Probe 9 | 2.48 | -0.49 | 2.48 | 2.96 |
| Probe 10 | 2.37 | -0.29 | 2.37 | 2.84 |
| Probe 11 | 1.72 | -0.65 | 1.72 | 2.06 |
| Probe 12 | 2.43 | -0.44 | 2.43 | 2.91 |
| Probe 13 | 2.23 | -0.45 | 2.23 | 2.67 |
| Probe 14 | 1.78 | -0.73 | 1.78 | 2.13 |
| Probe 15 | 2.55 | -0.57 | 2.55 | 3.06 |
| Probe 16 | 2.80 | -0.42 | 2.80 | 3.36 |
| Probe 17 | 2.44 | -0.67 | 2.44 | 2.92 |
| Probe 18 | 3.08 | -0.58 | 3.08 | 3.69 |
| Probe 19 | 3.14 | -0.50 | 3.14 | 3.75 |
| Probe 20 | 2.80 | -0.54 | 2.80 | 3.35 |
| Probe 21 | 2.77 | -0.67 | 2.77 | 3.32 |
| Probe 22 | 2.73 | -0.61 | 2.73 | 3.26 |
| Probe 23 | 2.48 | -0.79 | 2.48 | 2.96 |
| Probe 24 | 2.79 | -0.83 | 2.79 | 3.34 |
| Probe 25 | 3.14 | -0.69 | 3.14 | 3.75 |
| Probe 26 | 2.45 | -0.95 | 2.45 | 2.93 |
| Probe 27 | 2.67 | -0.87 | 2.67 | 3.19 |
| Probe 28 | 2.46 | -0.85 | 2.46 | 2.94 |
| Probe 29 | 2.38 | -1.02 | 2.38 | 2.85 |
| Probe 30 | 2.26 | -0.94 | 2.26 | 2.71 |
| Probe 31 | 2.15 | -0.91 | 2.15 | 2.57 |
| Probe 32 | 2.61 | -1.04 | 2.61 | 3.12 |
| Probe 33 | 2.28 | -1.08 | 2.28 | 2.73 |
| Probe 34 | 3.12 | -0.86 | 3.12 | 3.73 |
| Probe 35 | 2.57 | -0.99 | 2.57 | 3.08 |
| Probe 36 | 1.95 | -1.10 | 1.95 | 2.33 |


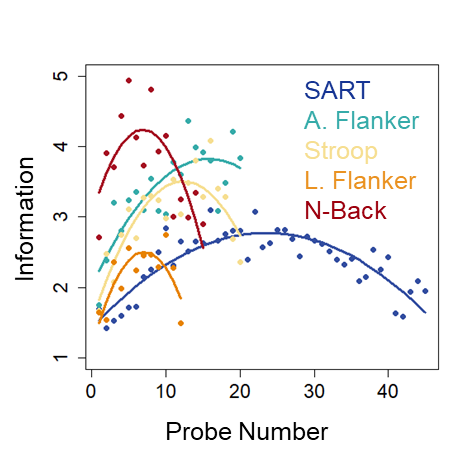


**Supplementary Figure 1:** Information is shown from nominal response models for data from Kane et al. (2016). Information is shown for tasks across sequential probes. Solid lines depict the polynomial quadratic trajectories of the values.


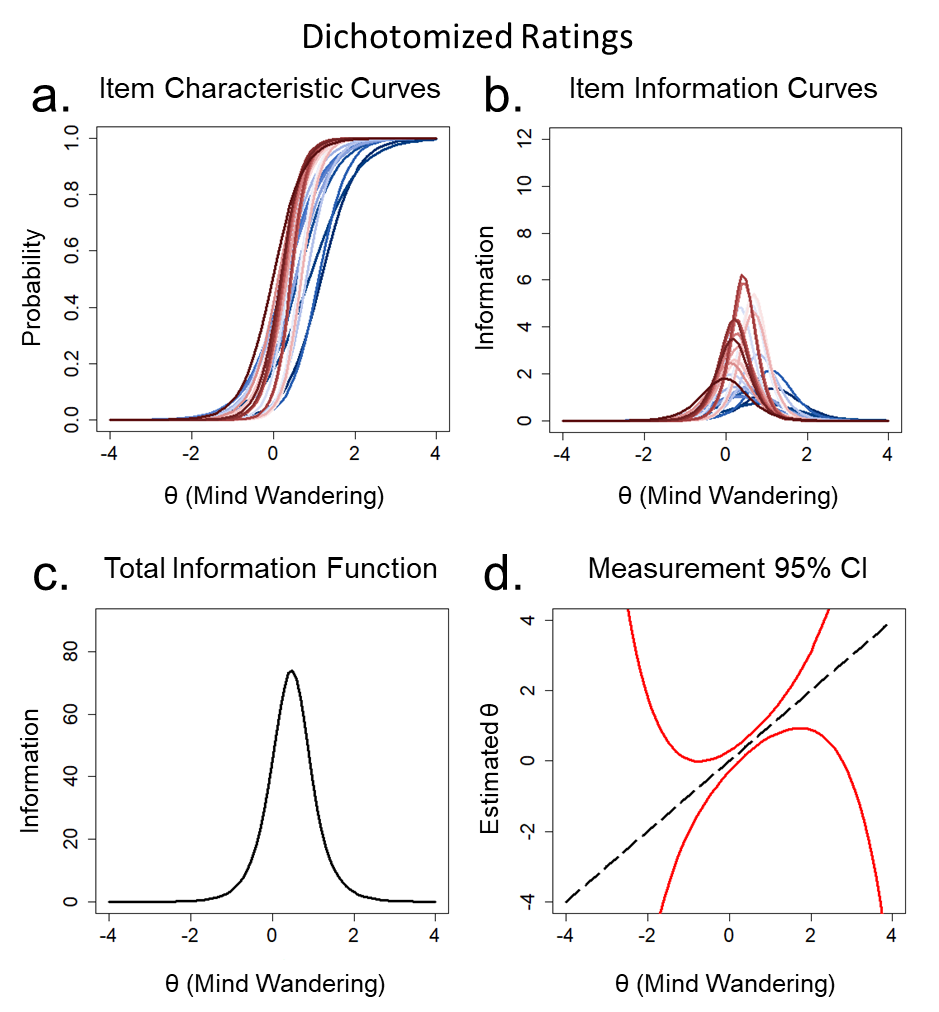


**Supplementary Figure 2:** Panels a–d reflect item response characteristics of continuous data from Zanesco et al. (2020) converted to dichotomized responses. Ratings > 1 were categorized as “off-task”. **Panel a** displays item characteristic curves based on the 2PL model shown for the probes when dichotomized. Curves represent the probability of a participant endorsing an “off-task” response at all levels of trait mind wandering (θ). **Panel b** displays item information curves for all probes. **Panel c** displays the total information. **Panel d** displays measurement precision of estimates of trait mind wandering (θ) shown based on the total information. The red line depicts the 95% CI around θ estimates at all levels of θ.

**
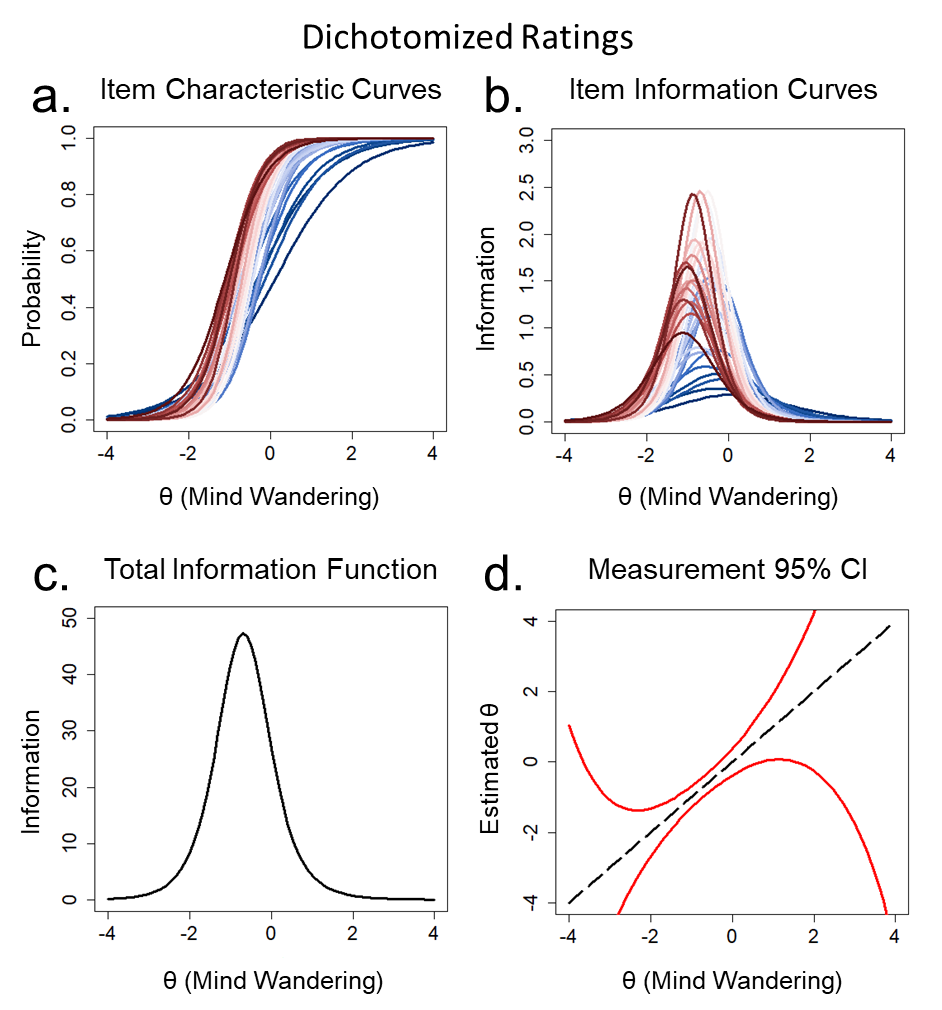
**

**Supplementary Figure 3:** Panels a–d reflect item response characteristics of continuous data from Goller et al. (2020) converted to dichotomized responses. Ratings > 1 were categorized as “off-task”. **Panel a** displays item characteristic curves based on the 2PL model shown for the probes when dichotomized. Curves represent the probability of a participant endorsing an “off-task” response at all levels of trait mind wandering (θ). **Panel b** displays item information curves for all probes. **Panel c** displays the total information. **Panel d** displays measurement precision of estimates of trait mind wandering (θ) shown based on the total information. The red line depicts the 95% CI around θ estimates at all levels of θ.
